# Stochastic Cytokine Expression Induces Mixed T Helper Cell States

**DOI:** 10.1371/journal.pbio.1001618

**Published:** 2013-07-30

**Authors:** Miaoqing Fang, Huangming Xie, Stephanie K. Dougan, Hidde Ploegh, Alexander van Oudenaarden

**Affiliations:** 1 Department of Biological Engineering, Massachusetts Institute of Technology, Cambridge, Massachusetts, United States of America; 2 Department of Physics, Massachusetts Institute of Technology, Cambridge, Massachusetts, United States of America; 3 Whitehead Institute for Biomedical Research, Cambridge, Massachusetts, United States of America; 4 Department of Biology, Massachusetts Institute of Technology, Cambridge, Massachusetts, United States of America; 5 Hubrecht Institute, Royal Netherlands Academy of Arts and Sciences and University Medical Center Utrecht, Utrecht, The Netherlands; University of Pennsylvania, United States of America

## Abstract

During eukaryotic development, the induction of a lineage-specific transcription factor typically drives differentiation of multipotent progenitor cells, while repressing that of alternative lineages. This process is often mediated by some extracellular signaling molecules, such as cytokines that can bind to cell surface receptors, leading to activation and/or repression of transcription factors. We explored the early differentiation of naive CD4 T helper (Th) cells into Th1 versus Th2 states by counting single transcripts and quantifying immunofluorescence in individual cells. Contrary to mutually exclusive expression of antagonistic transcription factors, we observed their ubiquitous co-expression in individual cells at high levels that are distinct from basal-level co-expression during lineage priming. We observed that cytokines are expressed only in a small subpopulation of cells, independent from the expression of transcription factors in these single cells. This cell-to-cell variation in the cytokine expression during the early phase of T helper cell differentiation is significantly larger than in the fully differentiated state. Upon inhibition of cytokine signaling, we observed the classic mutual exclusion of antagonistic transcription factors, thus revealing a weak intracellular network otherwise overruled by the strong signals that emanate from extracellular cytokines. These results suggest that during the early differentiation process CD4 T cells acquire a mixed Th1/Th2 state, instructed by extracellular cytokines. The interplay between extracellular and intracellular signaling components unveiled in Th1/Th2 differentiation may be a common strategy for mammalian cells to buffer against noisy cytokine expression.

## Abstract 2

During early differentiation of T helper cells, stochastic cytokine expression triggers the co-expression of antagonistic transcription factors at high levels, buffered by the interplay between extracellular and intracellular signaling components.

## Author Summary

During the development of a multicellular organism, the progenitor cells, which have the potential to become any of several different cell lineages with specialized functions, commit and differentiate into one particular lineage. This differentiation of progenitors is driven by the induction of lineage-specific transcription factors, molecules that regulate gene expression. This process is often mediated by extracellular signaling molecules, including a class of molecules called cytokines that can bind to cell surface receptors, activating and/or repressing transcription factors. Here we explored the early differentiation of naive T helper (Th) cells, an important class of T lymphocytes that help effector immune cells to defend the body against various pathogens. We measured both mRNA and protein levels of cytokines and transcription factors in individual cells. In particular, mRNA levels were measured with single-molecule resolution. Contrary to the expression of only one set of lineage-specific transcription factors, we observed ubiquitous high-level co-expression of antagonistic transcription factors in individual cells. We found that cytokines are expressed only in a small subpopulation of cells, independent from the expression of transcription factors in individual cells. When cytokine signaling is inhibited, each cell expressed only one of the antagonistic transcription factors at high levels. This reveals a weak intracellular network that is otherwise overruled by the strong signals that emanate from extracellular cytokines. These results suggest that during the early differentiation process T helper cells acquire a mixed Th1/Th2 state, instructed by extracellular cytokines. The interplay between extracellular and intracellular signaling components unveiled in Th1/Th2 differentiation may be a common strategy for mammalian cells to buffer against noisy cytokine expression.

## Introduction

A multipotent progenitor cell can differentiate into a particular lineage by turning on the expression of a lineage-specific transcription factor, which coordinates the expression of a defined set of target genes. Numerous examples of such toggle-switch-like cell fate decisions have been observed in the differentiation of hematopoietic cells [1]. For example, common myeloid progenitor cells differentiate into granulocyte-monocyte progenitor versus megakaryocyte-erythrocyte progenitor cells based on expression of PU.1 versus Gata1 [2]; naive CD4 T cells differentiate into Th1 versus Th2 driven by the expression of Tbet or Gata3 [3]–[6]. Antagonistic transcription factors are therefore believed to be expressed exclusively in the pertinent cell types, or co-expressed at basal levels in hematopoietic progenitors prior to commitment to “prime” the cells for rapid deployment of transcription factors to execute a particular lineage program [7]. For instance, common myeloid progenitors can co-express low levels of PU1 and GATA1 during lineage priming [8]–[12], though their expression is mutually exclusive in the fully committed state [7].

In addition to transcription factors that reside within the cell, the signaling network governing cell differentiation often comprises extracellular components, such as cytokines that can bind to cell surface receptors leading to activation and/or repression of transcription factors. In many previous studies, where the goal has been attaining a relatively homogenous population of differentiated cells, high concentrations of cytokines were added to the culture media to bias the cellular decision process toward one particular cell fate [2],[3],[6],[13].

In this work, we studied gene regulation during the early stage of cell differentiation to delineate the interplay between extracellular cytokines and intracellular transcription factors in single cells, using CD4 T helper cells as a model system. Contrary to previous studies where cellular fate was biased artificially [2],[3],[6],[13], we sought to avoid this bias by exploring the spontaneous differentiation of naive CD4 T cells in the absence of exogenously added cytokines.

**Figure 1:**
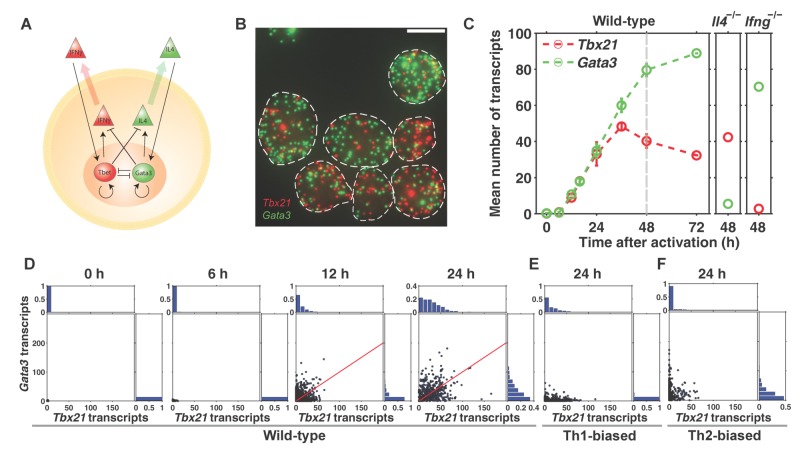
*Tbx21* and *Gata3* are transcribed simultaneously in individual CD4 T cells. (A) Current gene regulatory network proposed to govern Th1/Th2 differentiation. (B) Visualization of single transcripts of *Tbx21* (red) and *Gata3* (green) in individual CD4 T cells 24 h after activation. White dashed lines are boundaries of individual cells. Scale bar, 10 µm. (C) Mean counts of *Tbx21* and *Gata3* transcripts per cell as a function of activation time. (D) Scatter plots of *Tbx21* and *Gata3* transcripts in individual cells, with marginal distributions. The red line is the median line that divides data points into halves. Individual cells do not show mutual exclusion of *Tbx21* and *Gata3* expression. (E) Scatter plots of *Tbx21* and *Gata3* transcripts at 24 h in CD4 T cells treated with Th1-polarizing condition supplemented with 10 ng/ml IFNγ and IL12 and 10 µg/ml anti-IL4 antibody. (F) Scatter plots of *Tbx21* and *Gata3* transcripts at 24 h in CD4 T cells treated with Th2-polarizing conditions supplemented with 10 ng/ml IL4 and 10 µg/ml anti-IFNγ antibody. Error bars are s.e.m. of replicate experiments.

Tbet, encoded by *Tbx21*, is the master transcription factor of Th1 differentiation associated with production of the hallmark cytokine IFNγ [3], whereas Gata3 is the master transcription factor of Th2 differentiation associated with IL4 production [5]. In terminally differentiated individual CD4 T cells, the expression of *Tbx21* and *Gata3* is mutually exclusive [14],[15]. This is attributed to positive feedback loops and cross-inhibitory interactions in the regulatory network (Figure 1A). This network consists of two types of interactions: those that depend on cytokine signaling and those that are cytokine-independent and involve only intracellular players including transcription factors. Specifically, Tbet activates *Ifng* [16], and extracellular IFNγ can induce *Tbx21* via receptor signaling [17]. Tbet also induces itself independently of signaling via cytokine receptors [18]. Similarly, Gata3 activates *Il4* [19],[20] and extracellular IL4 can induce *Gata3* [21]. Furthermore, *Gata3* can be auto-induced independently of signaling via cytokine receptors [19],[22]. Finally, Tbet silences *Il4* [16], Gata3 silences *Ifng* [23],[24], and Tbet blocks the function of Gata3 through direct protein–protein interactions [25], leading to cross-inhibitory interactions.

To quantify the number of *Tbx21* and *Gata3* transcripts in activated CD4 T cells, we isolated total CD4^+^ cells from C57BL/6 mice. CD4 cells were then activated by culturing them in wells coated with anti-CD3 and anti-CD28 antibodies, in the absence of polarizing cytokines or neutralizing antibodies against cytokines, such that CD4 T cells would choose their cell fates without being biased. We performed single-molecule fluorescent *in situ* hybridization (smFISH) [26] combined with immunofluorescence to quantify transcripts and protein levels in individual cells (Figures S1 and S2).

## Results

### High-Level Co-expression of *Tbx21* and *Gata3* in Individual Cells

Without exogenously imposed Th1- or Th2-biasing cues, naive CD4 T cells, essentially expressing zero copies of *Tbx21* and *Gata3* transcripts, turned on expression of both *Tbx21* and *Gata3* simultaneously in individual cells after activation (Figure 1B–D). Simultaneous up-regulation of *Tbx21* and *Gata3* occurs very rapidly within 24 h, in contrast to co-expression of *Tbx21* and *Gata3* observed weeks after activation from naive cells followed by a reprogramming experiment [27]. Furthermore, distinct from basal co-expression in lineage priming [8]–[12], co-expression of *Tbx21* and *Gata3* are at high levels, such that the mean number of *Gata3* transcripts per cell at 48 h is comparable to fully differentiated Th2 cells [28].

To further assess the expression levels of *Tbx21* and *Gata3* that we observed under non-biased condition, we compared to cells that were treated under standard polarizing conditions with supplements of IFNγ and IL4 as well as neutralizing antibodies against opposing cytokines as previously described [16]. While polarized cells express *Tbx21* and *Gata3* in a mutually exclusive manner, we found that the expression of the up-regulated transcription factor is comparable to the high-level co-expression in cells under non-biased condition (Figure 1E,F), indicating that that the cells under non-biased condition produce IFNγ and IL4 by themselves. Furthermore, supplementing both IFNγ and IL4 into the non-biased cell culture did not increase the expression of *Tbx21* and *Gata3* further (unpublished data), indicating that the amount of IFNγ and IL4 that CD4 T cells produce has already reached saturation for signaling.

In addition, high-level co-expression of *Tbx21* and *Gata3* in individual cells is a robust phenomenon observed over a large range of seeding cell density (Figure S3). Interestingly, the median stoichiometry between *Tbx21* and *Gata3* expression was 1∶1 until 24 h after activation, but *Gata3* levels continued to increase after 24 h while *Tbx21* levels decreased (Figure S4). As activation time increases, the culture system presumably accumulates more Th2-favoring cytokines. Since most of the significant changes in gene expression occurred within this 48 h period, we focused our analyses on this period in subsequent experiments.

**Figure 2:**
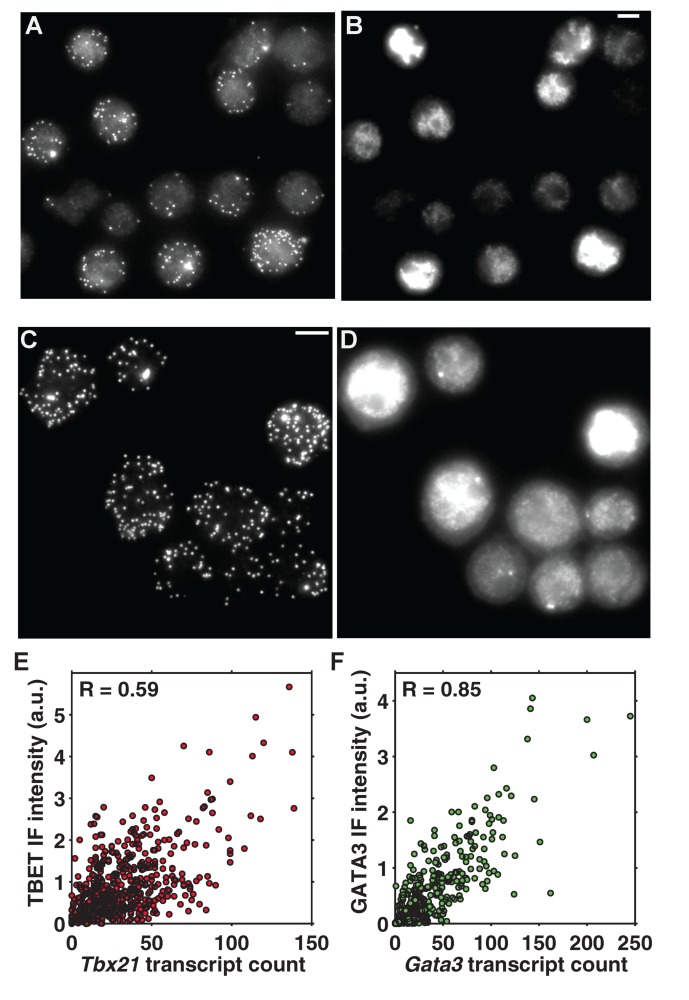
Transcript and protein levels exhibit strong positive correlations. (A, B) Visualization of single *Tbx21* transcripts by mRNA-FISH (A) simultaneously with protein levels by immunofluorescence (B) at 24 h after activation. (C, D) Visualization of single *Gata3* transcripts by mRNA-FISH (C) simultaneously with protein levels by immunofluorescence (D) at 24 h after activation. All scale bars are 10 µm. (E) Scatter plot of transcript counts versus protein levels for Tbet at 24 h, with a Pearson's correlation coefficient of 0.59 (*p*<10^−44^). (F) Scatter plot of transcript counts versus protein levels for Gata3 at 24 h, with a Pearson's correlation coefficient of 0.85 (*p*<10^−84^).

To demonstrate that transcript counts serve as a good proxy for protein levels, we performed immunofluorescence against Tbet or Gata3 simultaneously with smFISH. Transcript counts and protein levels showed strong correlations in individual CD4 T cells, with a Pearson's correlation coefficient *R* of 0.59 (*p*<10^−44^) for Tbet and 0.85 (*p*<10^−84^) for Gata3 (Figure 2). In addition, translational efficiency, measured by the ratio of immunofluorescence intensity over transcript count, remained constant as a function of activation time (Figure S5).

It is interesting to note that the expression of two other transcription factors Foxp3 and RORγT, which control the other two lineages of CD4 T helper cells [induced regulatory T helper cells (iTreg) and Th17], are not excluded from cells that express *Tbx21* and *Gata3* (Figure S6). We have shown that there is no strong positive or negative correlation between these four transcription factors (Figure S6). Therefore, during early stage of CD4 T helper cell differentiation, lack of mutual exclusivity does not only apply to Tbet and Gata3, but also to Foxp3 and RORγT.

### A Rare Cell Population Expresses the Cytokine Genes

We postulated that ubiquitous *Tbx21* and *Gata3* co-expression must be associated with both Th1 and Th2 cytokines produced by CD4 T cells upon activation [29], since no cytokines were supplied exogenously. We thus investigated the expression of cytokines in individual CD4 T cells. Among several Th1 and Th2 cytokines, we focused our effort on *Ifng* and *Il4*. In activated *Ifng* ^−/−^ mutant cells, we observed a 95% reduction in *Tbx21* expression (mean of 33 in wild-type versus 1.6 in *Ifng* ^−/−^ mutant at 24 h) and a 93% reduction in *Gata3* expression in activated *Il4* ^−/−^ mutant cells (mean of 35 in wild-type versus 2.3 in *Il4* ^−/−^ mutant at 24 h) (Figure S7) [30],[31]. Our results indicate that the hallmark cytokines *Ifng* or *Il4* alone is responsible for achieving more than 90% of the expression of *Tbx21* or *Gata3*. Therefore, any other cytokines minimally contribute to the expression of *Tbx21* or *Gata3*.

**Figure 3:**
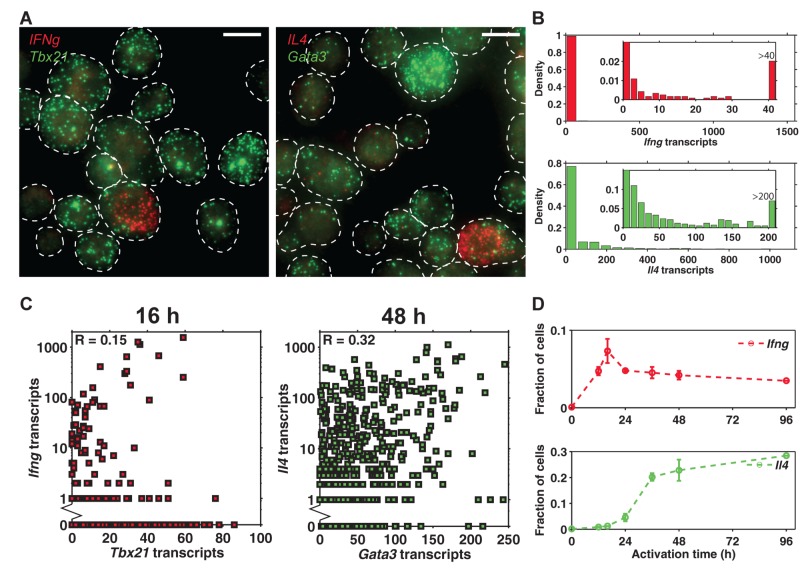
*Ifng* and *Il4* are expressed in a rare cell population and their levels show no significant correlation with *Tbx21* and *Gata3* expression. (A) Visualization of single transcripts of *Tbx21* and *Ifng*, and *Gata3* and *Il4* in individual CD4 T cells at 48 h. All scale bars are 10 µm. (B) Distribution of *Ifng* and *Il4* transcripts in individual CD4 T cells, with inset diagrams to better illustrate the fraction of cells that express non-zero copies of cytokines. (C) Scatter plots show a weak positive correlation between *Tbx21* and *Ifng* expression, or between *Gata3* and *Il4* expression. (D) Fraction of cells that express *Ifng* (defined as >20 transcripts) and that expressing *Il4* (defined as >50 transcripts) as a function of activation time. Error bars are s.e.m. of replicate experiments.

Current understanding of the gene regulatory network that governs Th1/Th2 differentiation would predict that *Ifng* or *Il4* transcripts are proportional to *Tbx21* or *Gata3* levels in individual cells. Surprisingly, we observed that *Ifng* and *Il4* were expressed only in a rare cell population. While the vast majority of cells were in the OFF state and contained essentially zero copies of *Ifng* or *Il4* transcripts, the rare ON cells expressed up to more than 1,000 transcripts, resulting in a dynamic expression range of three orders of magnitude (Figure 3A,B, Figure S8). In cells expressing more than 200 transcripts, we could not resolve individual mRNA molecules. Instead, we extrapolated the number of transcripts from the linear relationship between the total fluorescence and number of transcripts using cells with fewer transcripts (Figure S9). Complementary to previous studies reporting that the expression of cytokines in fully differentiated T helper cells is stochastic [32],[33], our work focused on the early phase of T helper cell activation, and showed that the cell-to-cell variation in the cytokine expression is significantly larger during the early phase than that in the fully differentiated state.

In addition, there was only a weak positive correlation between *Tbx21* and *Ifng* expression (*R* = 0.15, *p*<10^−6^), or between *Gata3* and *Il4* expression (*R* = 0.32, *p*<10^−11^) (Figure 3C). There was no negative correlation between *Gata3* and *Ifng* expression (*R* = 0.06, *p* = 0.04), or between *Tbx21* and *Il4* expression (*R* = 0.26, *p*<10^−9^) (Figure S10). In addition, we performed three-color smFISH experiments probing *Ifng* or *Il4* transcripts, while simultaneously detecting *Tbx21* and *Gata3*. Our data showed that the distributions of *Tbx21* and *Gata3* expression, conditioned on the number of *Ifng* or *Il4* transcripts, are invariant, indicating that *Tbx21* and *Gata3* expression do not correlate with *Ifng* or *Il4* expression (Figure S12). Therefore, the regulation of *Ifng* and *Il4* expression appears to be decoupled from the expression of *Tbx21* or *Gata3* levels in individual cells during early differentiation. It is interesting to note that *Ifng* and *Il4* are not the only cytokines showing such expression patterns, other cytokines such as Il13 are also expressed in a rare population with large variability in the expression level amongst individual cells (Figure S11).

We further examined the cytokine milieu surrounding the cells by quantifying the distance of each cell from its nearest cytokine producing neighbor. By plotting the cellular *Tbx21* and *Gata3* levels against the distance, we have found that there is no correlation between the two variables (Figure 3A, Figure S13), indicating that diffusion of cytokine is not rate-limiting and the cytokine milieu is well-mixed.

We also examined the correlation between the transcript and protein levels of cytokines. We found that cells that expressed a high number of cytokine transcripts also contained high levels of cytokine protein as detected by immunofluorescence. Transcriptionally inactive cells did not contain measurable levels of cytokine protein (Figure S14).

To ensure that the rare cytokine-producing cells were not non-naive CD4^+^ T cells, such as Natural Killer T (NKT) cells, we analyzed the expression of *Klrb1c*, which encodes the NKT cell marker NK1.1, and did not observe any NK1.1-expressing cells (Figure S15).

To ensure that the CD4 T cells we isolated did not contain effector memory cells, we compared naive CD4 T cells isolated by CD4^+^ positive selection with that isolated by negative selection using the mouse naive CD4 T cell isolation kit (Miltenyi). We first measured the CD44 protein levels in the isolated cells using immunofluorescence. We found that CD44 levels were low in naive T cells isolated by CD4^+^ positive selection, comparable to that isolated by the negative selection kit (Figure S15). We then cultured the naive T cells isolated by the negative selection kit, and found that there was no significant positive correlation between cytokine expression and CD44 levels in activated cells (Figure S16). In addition, the distribution of cytokine expression of the naive T cells isolated by the negative selection kit was identical to that selected by CD4^+^ positive selection (Figure S8). These results showed that rare cytokine-producing cells are not effector memory cells, but originated from bona fide naive cells.

Taken together, we conclude that a rare naive CD4 T cell population stochastically turns on *Ifng* or *Il4* independently of Tbet or Gata3 levels. These rare cells secrete cytokines into their surroundings and instruct other cells to ubiquitously express *Tbx21* and *Gata3*, and may thus play a pioneer role in determining the differentiation outcome of the entire cell population.

To test our hypothesis about the instructive role of cytokines on the expression of transcription factors, we analyzed their expression over the time course. While naive CD4 T cells contain essentially zero copies of cytokine transcripts, the fraction of *Ifng*-expressing cells increased from 0 to 16 h and decreased moderately afterwards, whereas the fraction of IL4-producing cells increased monotonously (Figure 3D). This pattern is consistent with the trend of the mean *Tbx21* and *Gata3* counts per cell (Figure 2C). In addition, it is worth noting that at the population level, the fraction of cells transcribing *Ifng* or *Il4* still positively correlates with the mean levels of *Tbx21* or *Gata3* transcripts, respectively, over time (correlation coefficient *R* = 0.35, *p* = 0.044 between *Tbx21* and *Ifng,* correlation coefficient *R* = 0.98, *p*<1.6×10^−44^ between *Gata3* and *Il4*). Therefore, these results indicate that cytokines are instructive on the expression of *Tbx21* and *Gata3*.

### Strength of Cytokine Signaling Predominates over Other Interactions

We revisited the signaling network governing Th1/Th2 choice during early CD4 T cell differentiation. Given that *Ifng* is transcribed in a rare pioneer cell population independently of *Tbx21* and *Gata3* levels, induction of *Ifng* by Tbet and repression by Gata3 can be neglected during the early stages of CD4 T cell differentiation, and a similar situation applies to *Il4*. Since *Tbx21* and *Gata3* are expressed simultaneously in individual cells without mutual exclusion, we postulated that the strength of receptor signaling mediated by cytokines must dominate over the intracellular network, namely self-activation and mutual inhibition of the transcription factors, which alone would lead to mutually exclusive expression of *Tbx21* and *Gata3*.

To demonstrate that the strength of cytokine signaling is dominant, we manipulated the amount of cytokine molecules available to cells by adding neutralizing antibodies. In the presence of saturating amounts of anti-IFNγ and anti-IL4, our data recapitulated the expression patterns of *Tbx21* and *Gata3* in *Ifng* ^−/−^ or *Il4* ^−/−^ cells, respectively (Figure S7), strongly indicating that this depletion strategy was specific (Figure S17). We postulated that any other cytokines minimally contribute to the expression of *Tbx21* or *Gata3*, and indeed our result showed that adding the antibody against another Th1 cytokine IL12 has no effect on *Tbx21* or *Gata3* expression (Figure S18).

**Figure 4:**
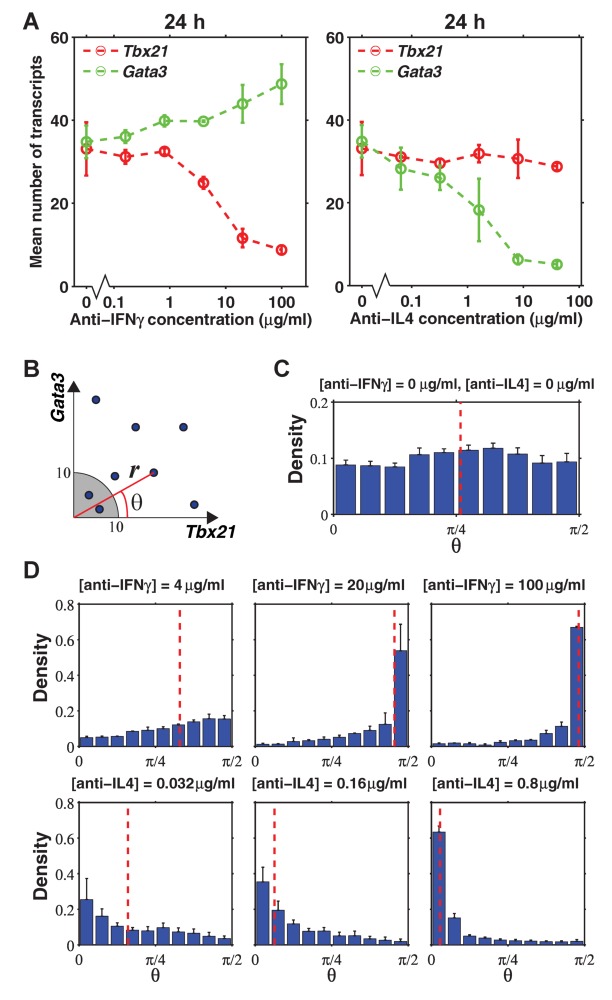
Inhibiting IFNγ and IL4 signaling down-regulates *Tbx21* and *Gata3*, respectively. (A) As the concentration of anti-IFNγ antibody increases, the mean number of *Tbx21* transcripts per cell decreases, while that of *Gata3* transcripts remains constant. The reverse is observed upon addition of anti-IL4 antibody. (B) Conversion of *Tbx21*-*Gata3* scatter plot into polar coordinates (*r*,*θ*), where *r* is the distance from the origin and computed by 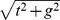, where *t* represents *Tbx21* and *g* represents *Gata3*, and *θ* is the angle with *x*-axis and computed by 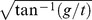 in the range 0≤*θ*≤π/2. (C) Distribution of *θ* for cells under non-biased condition is uniform, using the same data as Figure 1D. (D) Distribution of *θ* indicates that as concentration of anti-IFNγ antibody increases, the cells adopt larger *θ* (Th2-like state). The reverse is observed upon addition of anti-IL4 antibody. Red dashed lines show the medians of *θ*. All data shown are from cells at 24 h. Error bars are s.e.m. of replicate experiments.

Our results showed that sequestering a cytokine by the neutralizing antibody down-regulates the corresponding transcription factor but does not up-regulate the opposing transcription factor (Figure 1C, Figure 4A, Figure S17). For instance, at 24 h, *Tbx21* is down-regulated by 95% from a mean of 33 in wild-type to 1.6 in *Ifng* ^−/−^ mutant cells, but *Gata3* changes from a mean of 35 to 40 (Figure S7), which is a statistically insignificant change. Therefore, the role of extracellular cytokine signaling in specifying lineage choice is to up-regulate the corresponding transcription factor, rather than to repress that of the alternate lineage. In addition, the contribution of IFNγ to *Tbx21* expression is predominant, overshadowing the signaling strength of the intracellular components, namely self-activation and mutual inhibition of the transcription factors.

### A Polar-Coordinate Representation of the Data

Our results also show that down-regulation of the corresponding transcription factor could be modulated depending on the amount of neutralizing antibody (Figure 4A). To facilitate a quantitative interpretation of our data, we converted the *Tbx21*–*Gata3* scatter plot into polar coordinates of (*r*,*θ*), where *r* is the distance from the origin, which equals 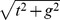, where *t* represents *Tbx21* and *g* represents *Gata3* transcript count. *θ* is the angle with the *x*-axis, which equals 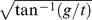 in the range 0≤*θ*≤π/2 (0°≤*θ*≤90°). Therefore, a small *θ* close to 0 means that a cell is Th1-like, and a large *θ* close to 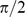 means Th2-like (Figure 4B). We excluded cells with *r*<10 (shaded region) in our analysis, because *θ* is not robust against small fluctuations in the number of transcripts in these cells. We then computed the distribution of *θ*, which gives us a measure on the cell fate bias. For instance, a uniform distribution of *θ* would mean that the cells can explore any intermediate state between Th1 and Th2 with no bias to a particular cell fate; whereas a “U”-shape distribution with probability density concentrat near 0 and π/2 would mean that the cells exhibit mutual exclusion by expressing a high level of only *Tbx21* or *Gata3*. To illustrate this polar-coordinate representation, we converted our data at 24 h under the non-biased condition and showed that *θ* follows an approximately uniform distribution, a hallmark of lacking mutual exclusion (Figure 4C, Figure S19). In other words, under non-biasing condition, CD4 T cells during early differentiation occupy any intermediate cell states between Th1 and Th2 with equal probability. We also showed that as the concentration of anti-IFNγ increases, the distribution of *θ* shifts toward π/2 (more Th2-like), whereas when the concentration of anti-IL4 increases, the distribution of *θ* shifts toward 0 (more Th1-like) (Figure 4D, Figures S20 and S21).

### A Model Governing the Early Activation of Naive CD4 T Cells

**Figure 5:**
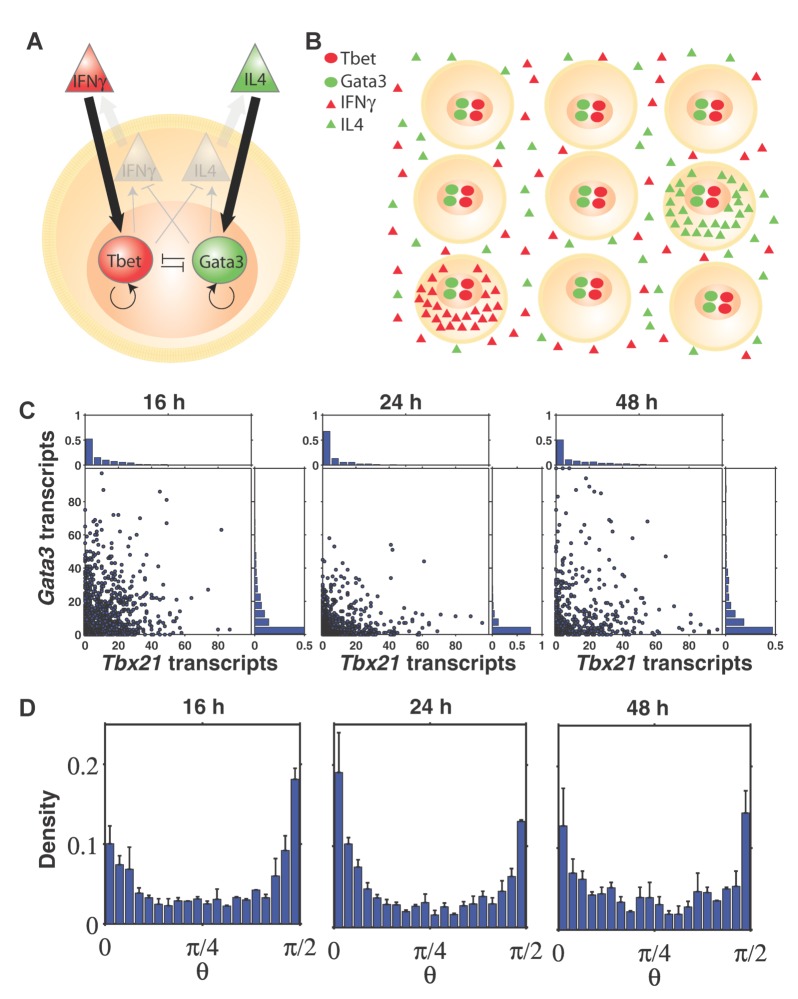
Sequestration of IFNγ and IL4 leads to mutually exclusive expression of *Tbx21* and *Gata3*. (A) Our model of the signaling network that governs Th1/Th2 differentiation. The thickness of arrows indicates the strength of interaction. The intracellular signaling network consists of all the interactions depicted in thin arrows. (B) Illustration of the CD4 T cell population during early activation. CD4 T cells are immersed in a well-mixed cytokine milieu established by the rare cytokine-expressing cells, leading to simultaneous and ubiquitous induction of *Tbx21* and *Gata3* expression in individual CD4 T cells. (C) Scatter plots showing down-regulation and mutual exclusion of *Tbx21* and *Gata3* transcripts in individual cells treated with both anti-IFNγ and anti-IL4 antibodies. (D) Distribution of *θ* shows that *θ* of most cells is very large (close to π/2) or small (close to 0) (same data as in Figure 5C). By two-sample Kolmogorov-Smirnov goodness-of-fit test, distribution of *θ* for cells under IFNγ and IL4 deprivation are significantly different from cells under non-biased condition, *p*<10^−11^ at 16 h, *p*<10^−19^ at 24 h, *p*<10^−54^ at 48 h. Error bars are s.e.m. of replicate experiments.

Taking the data together, we can explain the ubiquitous co-expression of *Tbx21* and *Gata3* under the non-biased condition: when CD4 T cells are exposed to both IFNγ and IL4 secreted by the rare cytokine producing cells, they up-regulate both *Tbx21* and *Gata3*. The key to the absence of mutually exclusive expression of *Tbx21* and *Gata3* is that cytokine signaling must predominate over self-activation of Tbet and Gata3 as well as mutual repression between Tbet and Gata3. This suggests that expression of *Tbx21* and *Gata3* is maintained at high levels by extracellular cytokine cues, with comparatively minimal effects from the intracellular signaling components (Figure 5A). Our data show that diffusion of cytokines is not rate-limiting (Figure S13), and we therefore propose that during early activation, CD4 T cells are bathed in a cocktail of well-mixed cytokine molecules produced by the rare pioneer cells, thus simultaneously inducing the expression of *Tbx21* and *Gata3* in individual CD4 T cells ubiquitously (Figure 5B).

According to our model, we hypothesized that by eliminating the extracellular IFNγ and IL4, only the intracellular signaling components will remain intact and should result in mutually exclusive expression of *Tbx21* and *Gata3* in individual cells. To verify this, we added both anti-IFNγ and anti-IL4. We tested multiple combinations of different concentrations of anti-IFNγ and anti-IL4 antibodies to find an optimum where the median of *θ* was close to π/4 (exactly in the middle of Th1 and Th2). We observed that under such conditions, *Tbx21* and *Gata3*, in addition to being down-regulated, are expressed in a mutually exclusive manner, such that the majority of cells are situated near the *Tbx21* or *Gata3* axis on the scatter plot and the distribution of *θ* falls into a “U”-shape distribution with higher density near 0 and π/2 (Figure 5C–D). We further explored the robustness of this phenomenon by culturing CD4 T cells under non-biased condition for 24 h first, allowing cells to first establish high-level coexpression of *Tbx21* and *Gata3*, and then added both anti-IFNγ and anti-IL4. Strikingly, the cells were still able to adopt mutually exclusive expression of *Tbx21* and *Gata3* (Figure S22). We therefore conclude that under simultaneous IFNγ and IL4 deprivation, only the comparatively weak intracellular signaling components that consist of the self-activation of Tbet and Gata3 as well as their mutual repression are functional, leading to mutually exclusive expression of *Tbx21* and *Gata3*.

## Discussion

Using CD4 T cells as a model of cell differentiation, we observed ubiquitous high-level co-expression of antagonistic transcription factors during the early stages of CD4 T cell differentiation under non-biased condition. CD4 T cells appear to produce a sufficient amount of IFNγ and IL4 for their own activation, such that *Tbx21* and *Gata3* are co-expressed at high levels. Strikingly, activation and cross-inhibition of *Ifng* and *Il4* expression appear to be decoupled from *Tbx21* and *Gata3* levels in individual cells (Figure 5A). Instead, *Ifng* and *Il4* are expressed by a rare population originated from bona fide naive cells, which do not appear to be contaminating NKT or memory CD4 T cells. We therefore postulate that these naive CD4 T cells stochastically turned on expression of *Ifng* or *Il4* and translate protein molecules ahead of the bulk population. These cytokine-producing cells, though rare, can direct the entire cell population into assuming one particular cell fate. By manipulating the amount of cytokine available to the cells, we demonstrated that signaling strength evoked by extracellular cytokines dominates over intracellular signaling components of self-activation and mutual inhibition. Therefore, put in the perspective of the debate on instruction versus selection model of immune cell differentiation [34], our results show that the role of extracellular cytokines is to instruct cells to up-regulate transcription factors during early stage of CD4 T cell differentiation. When IFNγ and IL4 are sequestered from the cells, only the intracellular signaling component is intact and hence the expression of *Tbx21* and *Gata3* is mutually exclusive.

### Stochastic Cytokine Expression in the Early Phase of T Help Cell Activation

**Figure 6:**
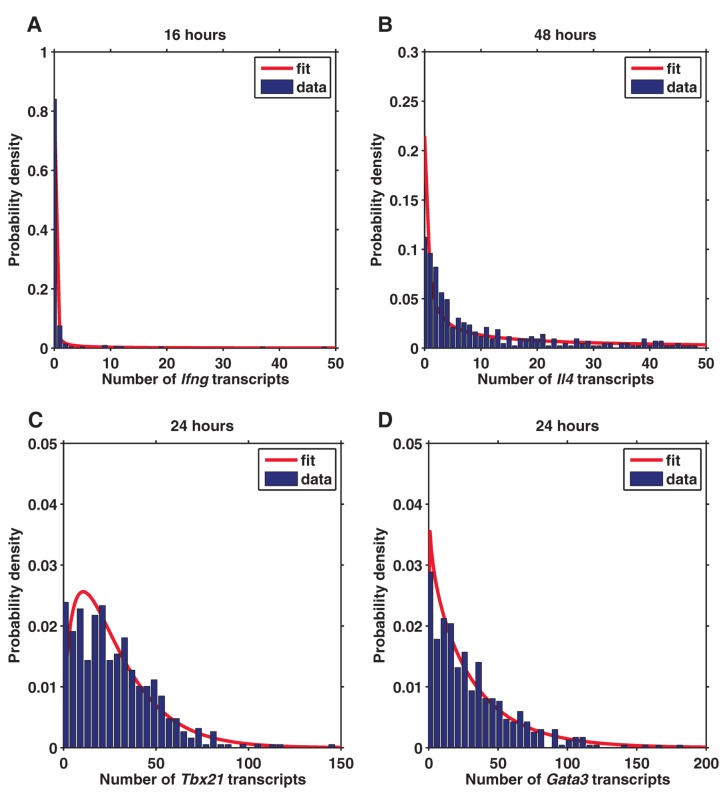
The distributions of cells expressing transcripts of *Ifng* (A), *Il4* (B), *Tbx21* (C), and *Gata3* (D). The data are fitted to Gamma distributions.

The large variability of cytokine expression between individual cells is very striking. Previous studies on unicellular organisms such as genetically identical populations of bacteria and yeast have observed lower variations in gene expression [35]–[38]. We were thus intrigued by the different mechanism of gene regulation in mammalian cells, where complex chromatin modeling that is not available to unicellular organisms may play an important role. Using a two-state model of transcription, where a gene needs to transition from an inactive to an active state before transcription can occur, the steady state of transcript density can be approximated to a Gamma distribution under the limiting case of short but infrequent bursts of mRNA synthesis [39]. We therefore fitted cells expressing cytokine transcripts to Gamma distributions. From the fitting, we can deduce that the average transcriptional burst size of *Ifng* is 159 and that of *Il4* is 176 (Figure 6A–B). Compared to the transcription factors, which have average burst size of 18 transcripts for *Tbx21* and 36 transcripts for *Gata3* (Figure 6C–D), the transcriptional bursts are much larger for cytokine genes, which are comparable to the very bursty 7×-tetO construct in the work of Raj et al. [39].

As cytokine molecules are produced and secreted to the cell culture media, a uniform cytokine milieu is established because diffusion of cytokine molecules is not rate-limiting (Figure S13), leading to up-regulation of transcription factors ubiquitously. It is interesting to note that although the production of cytokine molecules is highly heterogeneous amongst cells, the expression of transcription factors as a read-out is less variable because of averaging effect from mixing cytokine in the extracellular environment. The interplay between extracellular cytokines and intracellular transcription factors may be a common strategy for mammalian cells to buffer transcriptional noise that is otherwise intrinsic to the cells.

While cytokine expression appears to be decoupled from transcription factors in individual cells, we wondered how cytokine expression is regulated at the population level—for instance, how a cell population turns on IFNγ but not IL4 when cultured under Th1-favoring conditions with supplement of antibody against IL4. We quantified the expression of *Ifng* in cells treated with anti-IFNγ and the expression of *Il4* in cells treated with anti-IL4. We found that the number of cytokine-expressing cells and hence the mean of cytokine transcripts decreased when neutralizing antibody is added to the cell culture (Figure S23). Therefore, when cytokines are sequestered, not only the respective transcription factor gets down-regulated, but the expression of the cytokine itself is also down-regulated.

This observation suggests that although the expression of a cytokine is not positively correlated with the expression of its respective transcription factor in individual cells, the expression of cytokine in the entire cell population is still in concert with the expression level of the transcription factor. We postulate that transcription factor may be largely responsible for de-condensing the cytokine locus during early activation of CD4 T cells. While switching an inactive gene to the active state is a stochastic process in individual cells, the average of gene activation events is still deterministically controlled by the amount of transcription factors. As cell differentiation progresses, the activation of cytokine genes eventually becomes more ubiquitous and depends on the local concentration of active transcription factors, leading to higher positive correlation between a cytokine gene and the respective transcription factor in fully differentiated cells [27],[33],[40],[41].

In the light of our work, it will be interesting to delineate the underlying molecular mechanisms governing cytokine gene expression. In addition, given sufficient technological advances, it will be interesting to perform time-lapse experiments to track stochastic cytokine expression in individual cells over a time-course to visualize how these rare cytokine-producing cells arise and evolve over time. It will also be helpful to study the single-cell transcriptome of these cells to quantify how different these cells are from other cells. Insights from such experiments will shed light on the interplay between extracellular cytokines and the intracellular transcription factor on the fate specification of single cells. We note that mixed Th1–Th2 phenotypes were also observed concurrently by two other groups, using different experimental approaches [42],[43].

## Materials and Methods

### Strains of Mice Used

Experiments on wild-type cells were from C57BL/6 mice; experiments on *Il4* ^−/−^ cells were from B6.129P2-*Il4^tm1Cgn^*/J mice; experiments on *Ifng* ^−/−^ cells were from B6.129S7-*Ifng^tm1Ts^*/J mice. C57BL/6, *Ifng* ^−/−^, and *Il4* ^−/−^ mice were obtained from Jackson labs. All animals were housed at the Whitehead Institute for Biomedical Research and were maintained according to guidelines approved by the Massachusetts Institute of Technology (MIT) Committee on Animal Care.

### Cell Culture

Spleens and lymph nodes of mice aged from 6 wk to 2 mo were removed, suspended in PBS supplement with 2% FCS, and gently homogenized through a nylon mesh. Red blood cells were lysed with ammonium chloride solution (StemCell Technologies). CD4^+^ cells were isolated by MACS purification using the CD4 microbeads (Miltenyi Biotec) in all experiments except those that explicitly mentioned negative selection. In experiments where cells were selected by depletion, MACS CD4^+^ T cell isolation kit II was used. The medium used throughout the cell cultures was RPMI supplemented with 10% FCS, 2 mM L-glutamine, 1% penicillin, and streptomycin.

Cells were seeded into eight-well Lab-tek 1.0 coverglass chambers that had been coated with a mixture of anti-CD3 (15 µg/ml, clone 17A2) and anti-CD28 (15 µg/ml, clone 37.51) antibodies for at least 3 h, at 1,000,000 cells per well in a total volume of 0.5 ml, except one control experiment that explicitly mentioned 250,000 cells per well. The following neutralizing antibodies were used: IFNγ antibody (clone R4-6A2), IL4 antibody (clone BVD4-1D11), and IL12 antibody (clone C17.8). Cells were cultured at 37°C, 5% CO_2_. The first refresh of culture media occurred at 48 h, after which media was refreshed every 24 h. In experiments with Th1 polarization, 10 ng/ml IFNγ and IL12 and 10 µg/ml anti-IL4 antibody were supplemented in the media; in experiments with Th2 polarization, 10 ng/ml IL4 and 10 µg/ml anti-IFNγ antibody were supplemented in the media.

### Single-Molecule Fluorescence In Situ Hybridization (smFISH)

We performed smFISH on the T cells and counted the mRNAs in individual cells as described previously [26],[28]. Harvested T cells were fixed in PBS buffer with 3.7% formaldehyde for 10 min. After fixation, the cells were washed twice with PBS, permeabilized in 70% ethanol for at least 2 h, and stored at 4°C. The T cells were hybridized in the same glass chamber as cell culture. After the 70% ethanol was aspirated, the samples were washed in a solution of 25% formamide and 2×SSC for 5 min. After the wash buffer was aspirated, 100 µl of hybridization solution containing labeled DNA probes in 2×SSC, 1 mg/ml BSA, 10 mM VRC, 0.5 mg/ml Escherichia coli tRNA, and 0.1 g/ml dextran sulfate, with 25% formamide, were added to the sample and incubated overnight at 30°C. The next day, the samples were washed twice by adding 1 ml of wash solution consisting of 25% formamide and 2×SSC. For each wash, the sample was incubated in wash solution for 30 min. Then, the sample was resuspended in 2×SSC buffer. The sequences of FISH probes are available upon request.

### Immunofluorescence

To simultaneously visualize mRNA and protein levels in cells, we performed immunofluorescence after FISH protocol. The cells were incubated with 2×SSC, 0.2% Triton X-100, 5 mg/ml BSA, and fluorescent antibodies for 3 h at 4°C. Where a secondary antibody is required, the samples were incubated with 2×SSC, 0.2% triton X-100, 5 mg/ml BSA, and the secondary antibody for 1 h at 4°C. The cells were then washed by incubating with 2×SSC, 0.2% triton X-100, 5 mg/ml BSA for 1 h at 4°C. Tbet antibody is clone 4B10; Gata3 antibody is clone L50-823; IFNγ antibody is polyclonal (AMC4034, Invitrogen) and a secondary goat-anti-rabbit antibody (A11034, Invitrogen) is used. We test multiple IL4 antibodies for immunofluorescence, but none of them gave satisfactory signal-to-noise ratio.

### Image Acquisition

For imaging, the samples were soaked in glucose oxidase (glox) anti-fade solution, which contains 10 mM Tris (pH 7.5), 2×SSC, 0.4% glucose, supplemented with glucose oxidase and catalase. A coverslip was put over the sample. All images were taken with a Nikon Ti-E inverted fluorescence microscope equipped with a 100× oil-immersion objective and a Photometrics Pixis 1024 CCD camera using MetaMorph software (Molecular Devices, Downington, PA). Stacks of images were taken automatically with 0.4 microns between the z-slices.

### Image Analysis

To segment the T cells, a marker-guided watershed algorithm was used. Briefly, cell boundaries were obtained by running an edge detection algorithm on the bright-field image of the cells. To generate markers for watershed algorithm, the centroid of the region enclosed by individual cell boundaries is computed. A marker-guided watershed algorithm is then run on the distance transformation of the cell boundaries, using the markers located within the cell boundaries. The resultant cell segmentation image is then manually curated for occasional mis-segmentations.

To quantify the number of RNA molecules in each cell, a log filter is run over each optical slice of the image stack to enhance signals. A threshold is taken on the resultant image stack to pick up mRNA spots. The locations of mRNA spots are then taken to be the regional maximum pixel value of each connected region. The number of mRNA spots located within the cell boundaries of an individual cell can thus be quantified.

To quantify fluorescence signal in each cell, an optical slice corresponding to the central plane of the cells is analyzed. For each image, which covers up to 100 correctly segmented cells, the mean fluorescence per pixel of each cell is computed. The minimum of mean fluorescence is taken to be the background. Then for each cell in the image, the total fluorescence of the cell is computed as the sum of the fluorescence at each pixel subtracting the background. If this value is negative, zero is used instead.

## Supporting Information

Figure S1 **Segmentation of cells using bright-field images.** The left panel is a bright-field image of cultured Th cells. The right panel is the segmented image, using custom software written in MATLAB.
(TIF)

Figure S2 **Image analysis of mRNA spots.** The left panel is a fluorescent image showing *Tbx21* (red) and *Gata3* (green) transcripts in Th cells. The right panel is the processed image showing each individual mRNA transcript as a single bright red or green pixel. Scale bar, 10 µm.
(TIF)

Figure S3 **Scatter plots of** ***Tbx21*** **and** ***Gata3*** **transcripts in cell cultures of 250,000 cells per well at 24 h.** The cell density in this experiment is 4 times lower than that used in other experiments at 1,000,000 cells per well. It shows that the co-expression of *Tbx21* and *Gata3* transcripts in individual cells is robust over a range of cell densities.
(TIF)

Figure S4 **Scatter plots of** ***Tbx21*** **and** ***Gata3*** **transcripts in individual cells, with marginal distributions.** The red line divides the data set into two equal halves. The data show that no mutual exclusion of *Tbx21* and *Gata3* expression is observed in individual cells. The slope of the red line increases from 24 h to 48 h (compare with Figure 1D), indicating the ratio of *Gata3*–*Tbx21* increases from 24 h to 48 h.
(TIF)

Figure S5 **GATA3 immunofluorescence intensity versus** ***Gata3*** **transcript counts for cells at 24 h (left) and 48 h (right) after activation.** The red line is the least square fit of the data. The slope of 24-h data is 0.0032; that of 48-h data is 0.0038. The two experiments were performed on the same day with the same reagents and same microscope with same exposure time. This result shows that translational efficiency, indicated by the ratio of immunofluorescence intensity over transcript counts, remains constant as a function of activation time.
(TIF)

Figure S6
**There is no exclusivity in the expression of the four transcription factors, Foxp3, RORγT, Tbet, and Gata3, in individual cells.** (A) A fluorescent image of three-color smFISH probing *Tbx21* (blue), *Gata3* (green), and *Foxp3* (red) at 48 h. (*B*) A fluorescent image of two-color smFISH probing *Foxp3* (red) and *RORγT* (green) in T helper cells at 48 h. (C) Scatter plot of *Foxp3* versus *Tbx21* and *Gata3* transcripts at 48 h, where *Tbx21* and *Gata3* expression is condensed into a single axis computed by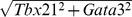. The Pearson's correlation coefficient is 0.14, indicating that the expression of *Foxp3* is not excluded from cells expressing *Tbx21* and *Gata3*. (D) Scatter plot of *Foxp3* and *RORγT* transcripts in T helper cells at 48 h (data collected on 627 cells). Pearson's correlation coefficient is 0.23, indicating that the expression of *Foxp3* and *RORγT* is not mutually exclusive.(TIF)

Figure S7 **Scatter plots of** ***Tbx21*** **and** ***Gata3*** **transcripts in individual cells of** ***Il4^−/−^*** **(A) and Ifng** ***^−/−^*** **(B) mice, with marginal distributions at 16 h, 24 h, and 48 h.** The expression of *Gata3* is down-regulated in *Il4^−/−^* mice. The expression of *Tbx21* is down-regulated in *Ifng^−/−^* mice.
(TIF)

Figure S8 **Fraction of cytokine-expressing cells at 24 h, in a control experiment that uses CD4 T cells purified by negative selection (MACS CD4^+^ T cell isolation kit II), in contrast to CD4 T cells purified by positive selection by CD4^+^ microbeads used in all the other experiments in this study.** Panel (a) shows the probability density of cells expressing *Ifng* transcripts; panel (b) shows the probability density of cells expressing *Il4* transcripts. We have shown that cultures of cells selected by negative selection also give rise to rare cells that stochastically express *Ifng* and *Il4* at high levels. Therefore, rare cytokine-expressing cells observed in Figure 3A,B are not an artifact of positive selection by CD4^+^ microbeads.
(TIF)

Figure S9
**Linear relationship exists between total fluorescent intensity of FISH and the computed mRNA transcripts in cells expressing fewer than 200 transcripts.** For the *Ifng* plot excluding points with more than 200 computed mRNA transcripts, Pearson's correlation coefficient = 0.86, 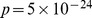; for the *Il4* plot excluding points with more than 200 computed mRNA transcripts, Pearson's correlation coefficient = 0.90, 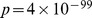. We can then extrapolate of the number of transcripts in highly expressing cells using the slope of the linear fit for cells expressing fewer than 200 transcripts.(TIF)

Figure S10
**Scatter plots showing that there is no negative correlation between** ***Gata3*** **and** ***Ifng*** **expression, with Pearson's correlation coefficient = 0.06,** 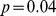 **, and that there is no negative correlation between** ***Tbx21*** **and** ***Il4*** **expression, with Pearson's correlation coefficient = 0.26,** 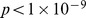 **.**
(TIF)

Figure S11
**The expression of** ***Il13*** **in activated Th cells has no strong correlation with the expression of Tbx21 or Gata3.** (A) A fluorescent image of three-color smFISH probing *Il13* (red), *Tbx21* (blue), and *Gata3* (green). (B) Scatter plot of the number of *Il13* transcripts versus *Tbx21* in CD4 T cells at 48 h with a Pearson's correlation coefficient *R* of 0.098 (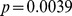). (C) Scatter plot of the number of *Il13* transcripts versus *Gata3* in CD4 T cells at 48 h with a Pearson's correlation coefficient *R* of 0.19 (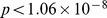).(TIF)

Figure S12 **The expression of** ***Tbx21*** **and** ***Gata3*** **does not depend on the expression of cytokines.** (A) Scatter plot of *Tbx21* versus *Gata3* color coded based on the expression of *Ifng*. (B) Scatter plot of *Tbx21* versus *Gata3* color coded based on the expression of *Il4*.
(TIF)

Figure S13 **The scatter plot of** ***Tbx21*** **(A) and** ***Gata3*** **(B) transcripts in individual cells versus the distance to the nearest** ***Ifng*** **-expressing (A) or** ***Il4*** **-expressing cell (B), which is defined as containing more than 20 transcripts of cytokines.** The position of each cell is computed as its centroid. It shows that the expression level of *Tbx21* and *Gata3* does not correlate with the distance from the near cytokine-expressing cell. Therefore, diffusion of cytokines from the source cells is not rate limited on the time scale of *Tbx21* and *Gata3* expression. Note that cells at 0 µm for the distance axis are the cytokine-expressing cells. Absence of cells between 0 µm and 7 µm is attributed to the fact that cell diameter is 7 µm, because cells are not overlapping in the mono-layer for imaging.
(TIF)

Figure S14 **Immunofluorescence together with single-molecule FISH on IFNγ shows that only cells expressing** ***Ifng*** **transcripts contain IFNγ protein.** Cytokine secretion was inhibited for 1 h to allow cytokine accumulation in these cells before harvesting. The top left panel is immunofluorescence image; the top right panel is single-molecule FISH image; the bottom left panel is the merge of immunofluorescence and single-molecule FISH; the bottom right panel is the bright field image. Scale bar, 10 µm.
(TIF)

Figure S15 **The cytokine-expressing cells are not NKT cells.** The left panel is the scatter plot of *Ifng* and *Klrb1c* transcripts showing that there is no significant positive correlation between *Ifng* and *Klrb1c*, Pearson's correlation coefficient = 0.095, *p* = 0.001, at 16 h after activation; the right panel shows the distribution of *Klrb1c* transcripts, indicating that *Klrb1c* expression is essentially OFF in all cells. Because *Klrb1c* encodes the marker NK1.1 for NKT cells, the cells expressing *Ifng* are not NKT cells that are not removed during magnetic sorting.
(TIF)

Figure S16
**Cytokine-expressing cells are not memory T cells.** (a) Scatter plot of CD44 immunofluorescence versus the number of *Ifng* or *Il4* transcripts shows that there is no significant positive correlation between CD44 levels and *Ifng* (correlation coefficient = 0.27, 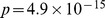 at 24 h; correlation coefficient = 0.094, 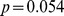 at 48 h) or *Il4* expression (correlation coefficient = 0.13, 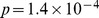 at 24 h; correlation coefficient = 0.0017, 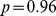 at 48 h). *Cd44* is a marker of memory T cells. Because cytokine-expressing cells do not preferentially express high levels of *Cd44* transcripts, they are not contaminating memory T cells that are not removed during magnetic sorting. (b) Probability density plot of CD44 immunofluorescence of naive T cells isolated by positive selection (CD4^+^ microbeads) or depletion (MACS CD4^+^ T cell isolation kit II). It shows that T cells isolated by positive selection, as used ubiquitously in this paper, are similar to T cells isolated by depletion, have low CD44 levels, and do not contain memory cells that are CD44^+^.(TIF)

Figure S17 **Scatter plots and marginal distributions showing that IFNγ antibody down-regulates** ***Tbx21*** **and IL4 antibody down-regulates** ***Gata3*** **at 24 h.**
(TIF)

Figure S18 **Scatter plots and marginal distributions of** ***Tbx21*** **and** ***Gata3*** **transcripts in individual cells treated with IL12 antibody, with the red line dividing data points into halves.** The left panel shows cells 16 h after activation; the right panel shows cells 24 h after activation. The result shows that anti-IL12 has no effect on the expression of *Tbx21* during early differentiation of Th cells.
(TIF)

Figure S19
**Distribution of** ***θ*** **under non-biased condition.** The left panel is 16 h after activation, where *θ* follows a uniform distribution. The right panel is 48 h after activation, where *θ* is skewed toward 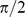, indicating cells become more Th2-like.(TIF)

Figure S20 **Distribution of** ***θ*** **at 16 h after activation.** Panel (a) shows that as concentration of anti-IFNγ antibody increases, the cells adopt larger *θ*. Panel (b) shows that as concentration of anti-IL4 antibody increases, the cells adopt smaller *θ*. Red lines are the medians of the *θ* distribution.
(TIF)

Figure S21 **Distribution of** ***θ*** **at 48 h after activation.** Panel (a) shows that as concentration of anti-IFNγ antibody increases, the cells adopt larger *θ*. Panel (b) shows that as concentration of anti-IL4 antibody increases, the cells adopt smaller *θ*. Red lines are the medians of the *θ* distribution.
(TIF)

Figure S22 **Distribution of** ***θ*** **at 48 h, where cells were not treated with any polarizing antibodies for the first 24 h, followed by the addition of both anti-IFNγ and anti-IL4 antibodies at 24 h.** It shows that the vast majority of cells adopt either very large or small *θ*, adopting either a Th1-like or Th2-like cell fate.
(TIF)

Figure S23 **Cytokine expression is down-regulated in the presence of neutralizing antibodies.** (A) Fraction of *Ifng*-expressing (defined as having >20 transcripts) decreases when anti-IFNγ is present in the cell culture. (B) Mean number of *Ifng* transcript per cell (defined as having >20 transcripts) decreases when anti-IFNγ is present in the cell culture. (C) Fraction of *Il4*-expressing (defined as having >50 transcripts) decreases when anti-IL4 is present in the cell culture. (D) Mean number of *Il4* transcript per cell (defined as having >50 transcripts) decreases when anti-IL4 is present in the cell culture.
(TIF)

## Abbreviations

FISH: fluorescence in situ hybridization
IFNγ: interferon gamma
IL: interleukin
NKT: natural killer cells
Th1: T helper cell type 1
Th2: T helper cell type 2
