# Supplementary figures and images for: Stochastic Cytokine Expression Induces Mixed T Helper Cell States
Source: PLoS Biol. 2013 Jul 30;11(7):e1001618. doi: 10.1371/journal.pbio.1001618 (PMC3728019; doi:10.1371/journal.pbio.1001618)

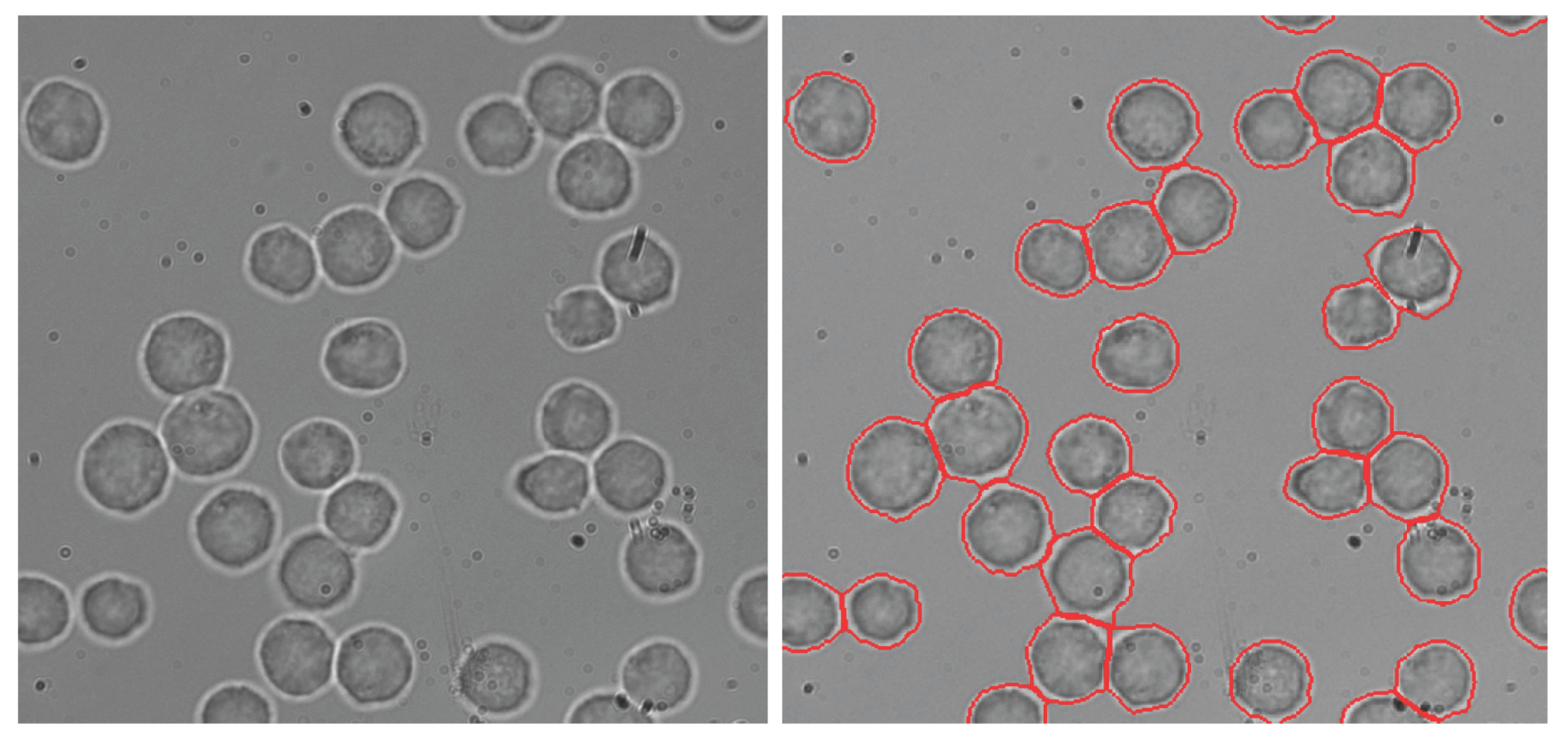

Supplement: Figure S1 — Segmentation of cells using bright-field images. The left panel is a bright-field image of cultured Th cells. The right panel is the segmented image, using custom software written in MATLAB. (TIF) [file pbio.1001618.s001.tif]

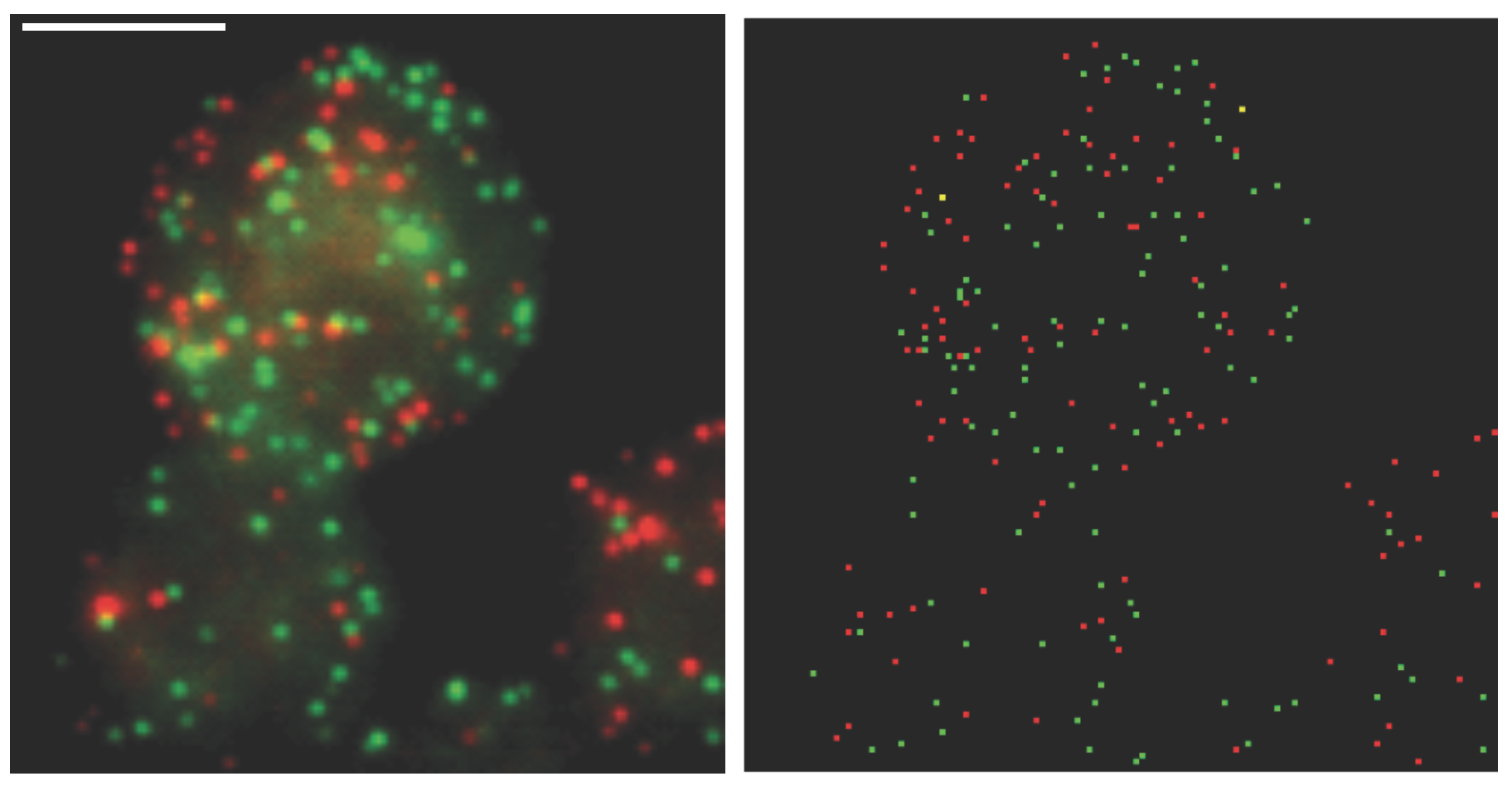

Supplement: Figure S2 — Image analysis of mRNA spots. The left panel is a fluorescent image showing Tbx21 (red) and Gata3 (green) transcripts in Th cells. The right panel is the processed image showing each individual mRNA transcript as a single bright red or green pixel. Scale bar, 10 µm. (TIF) [file pbio.1001618.s002.tif]

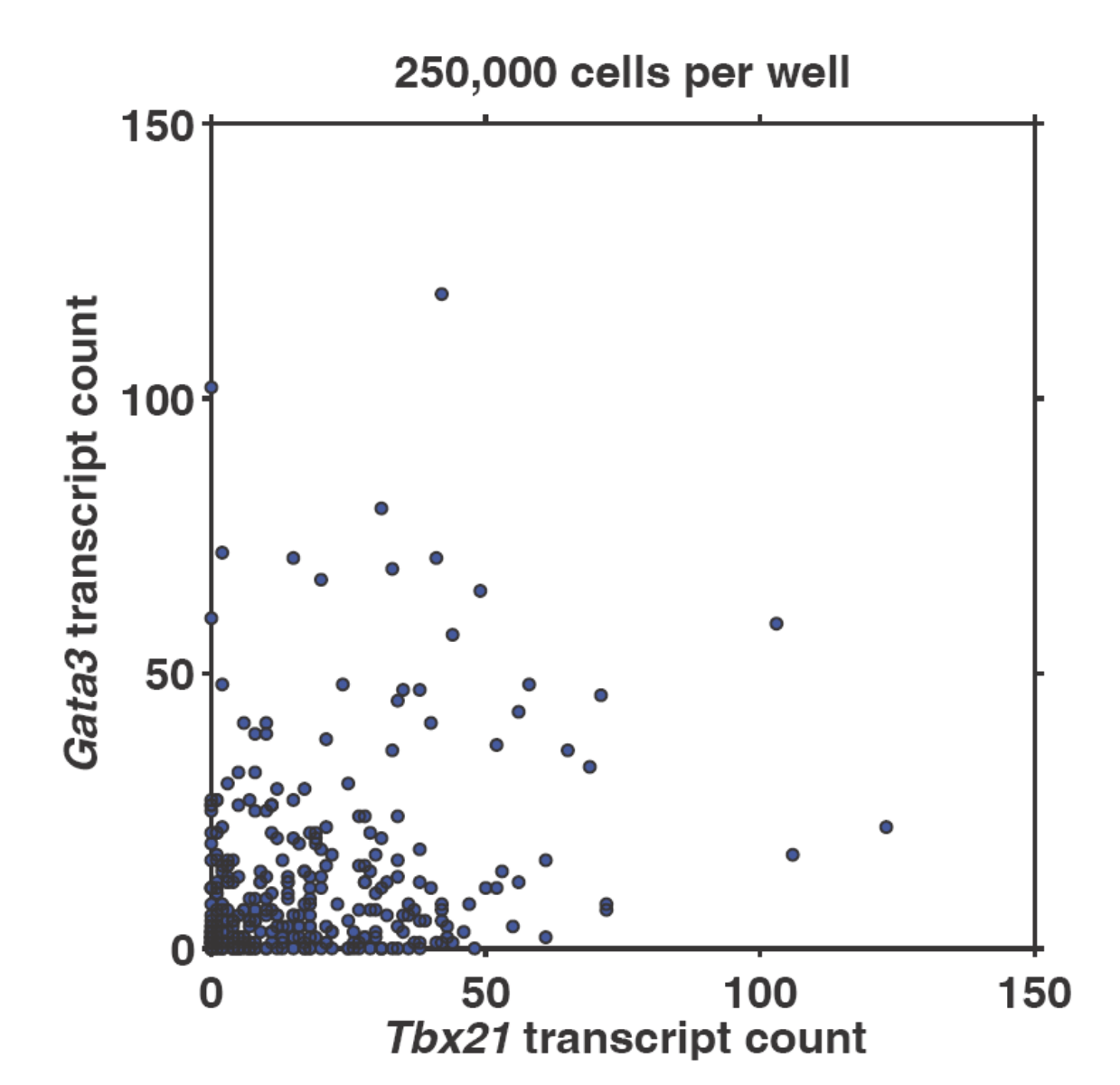

Supplement: Figure S3 — Scatter plots of Tbx21 and Gata3 transcripts in cell cultures of 250,000 cells per well at 24 h. The cell density in this experiment is 4 times lower than that used in other experiments at 1,000,000 cells per well. It shows that the co-expression of Tbx21 and Gata3 transcripts in individual cells is robust over a range of cell densities. (TIF) [file pbio.1001618.s003.tif]

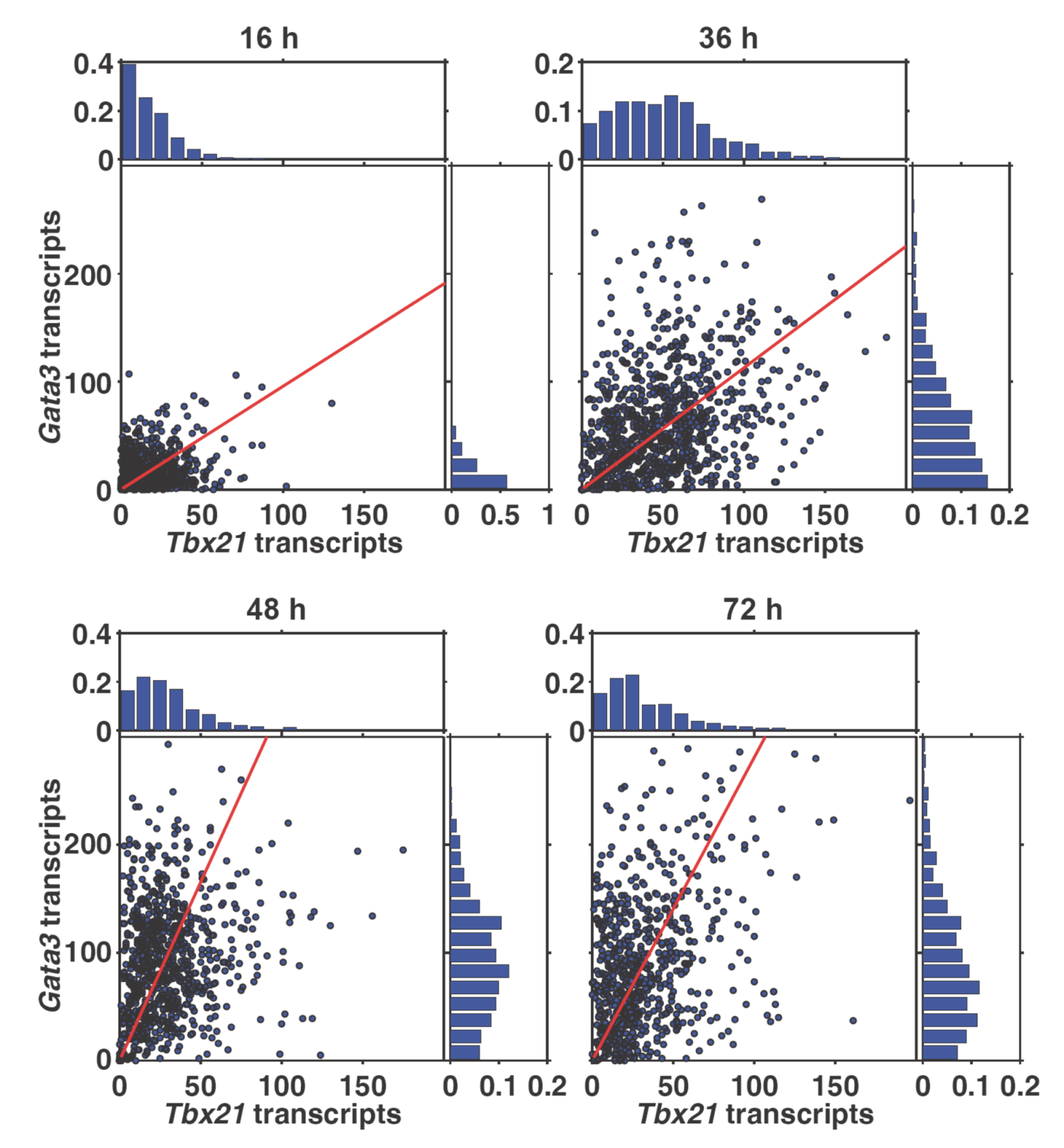

Supplement: Figure S4 — Scatter plots of Tbx21 and Gata3 transcripts in individual cells, with marginal distributions. The red line divides the data set into two equal halves. The data show that no mutual exclusion of Tbx21 and Gata3 expression is observed in individual cells. The slope of the red line increases from 24 h to 48 h (compare with Figure 1D), indicating the ratio of Gata3–Tbx21 increases from 24 h to 48 h. (TIF) [file pbio.1001618.s004.tif]

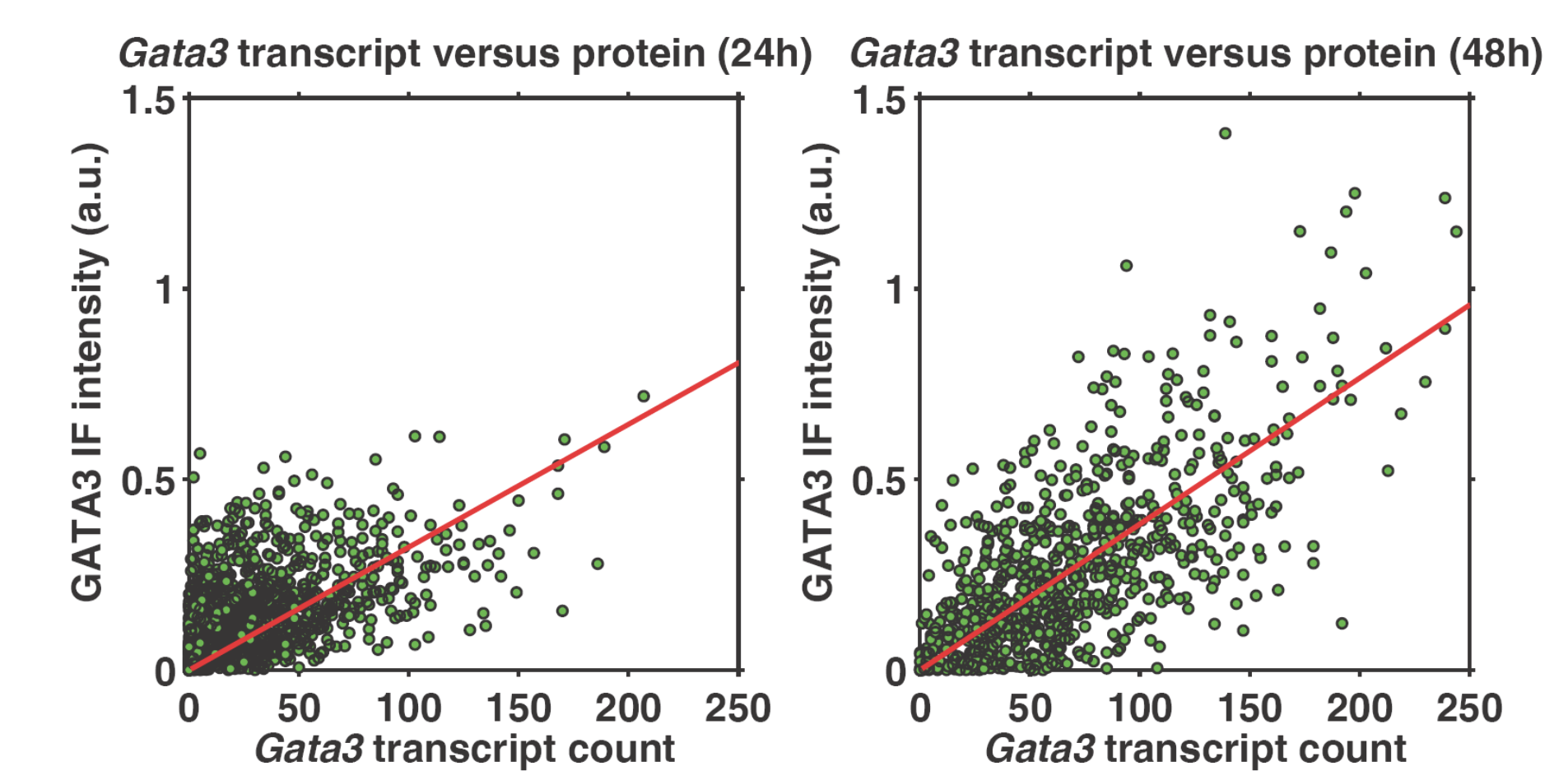

Supplement: Figure S5 — GATA3 immunofluorescence intensity versus Gata3 transcript counts for cells at 24 h (left) and 48 h (right) after activation. The red line is the least square fit of the data. The slope of 24-h data is 0.0032; that of 48-h data is 0.0038. The two experiments were performed on the same day with the same reagents and same microscope with same exposure time. This result shows that translational efficiency, indicated by the ratio of immunofluorescence intensity over transcript counts, remains constant as a function of activation time. (TIF) [file pbio.1001618.s005.tif]

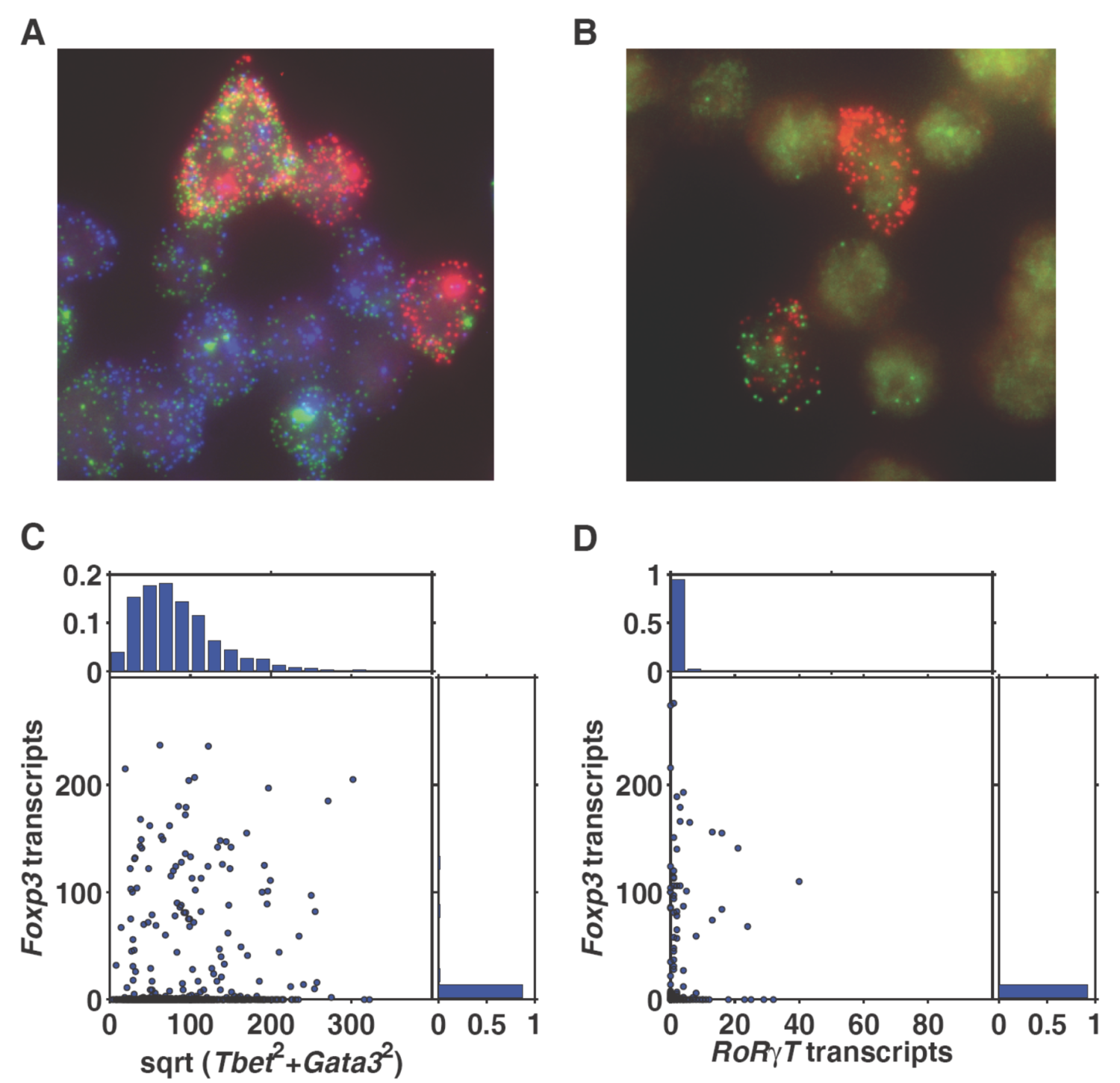

Supplement: Figure S6 — There is no exclusivity in the expression of the four transcription factors, Foxp3, RORγT, Tbet, and Gata3, in individual cells. (A) A fluorescent image of three-color smFISH probing Tbx21 (blue), Gata3 (green), and Foxp3 (red) at 48 h. (B) A fluorescent image of two-color smFISH probing Foxp3 (red) and RORγT (green) in T helper cells at 48 h. (C) Scatter plot of Foxp3 versus Tbx21 and Gata3 transcripts at 48 h, where Tbx21 and Gata3 expression is condensed into a single axis computed by. The Pearson's correlation coefficient is 0.14, indicating that the expression of Foxp3 is not excluded from cells expressing Tbx21 and Gata3. (D) Scatter plot of Foxp3 and RORγT transcripts in T helper cells at 48 h (data collected on 627 cells). Pearson's correlation coefficient is 0.23, indicating that the expression of Foxp3 and RORγT is not mutually exclusive. (TIF) [file pbio.1001618.s006.tif]

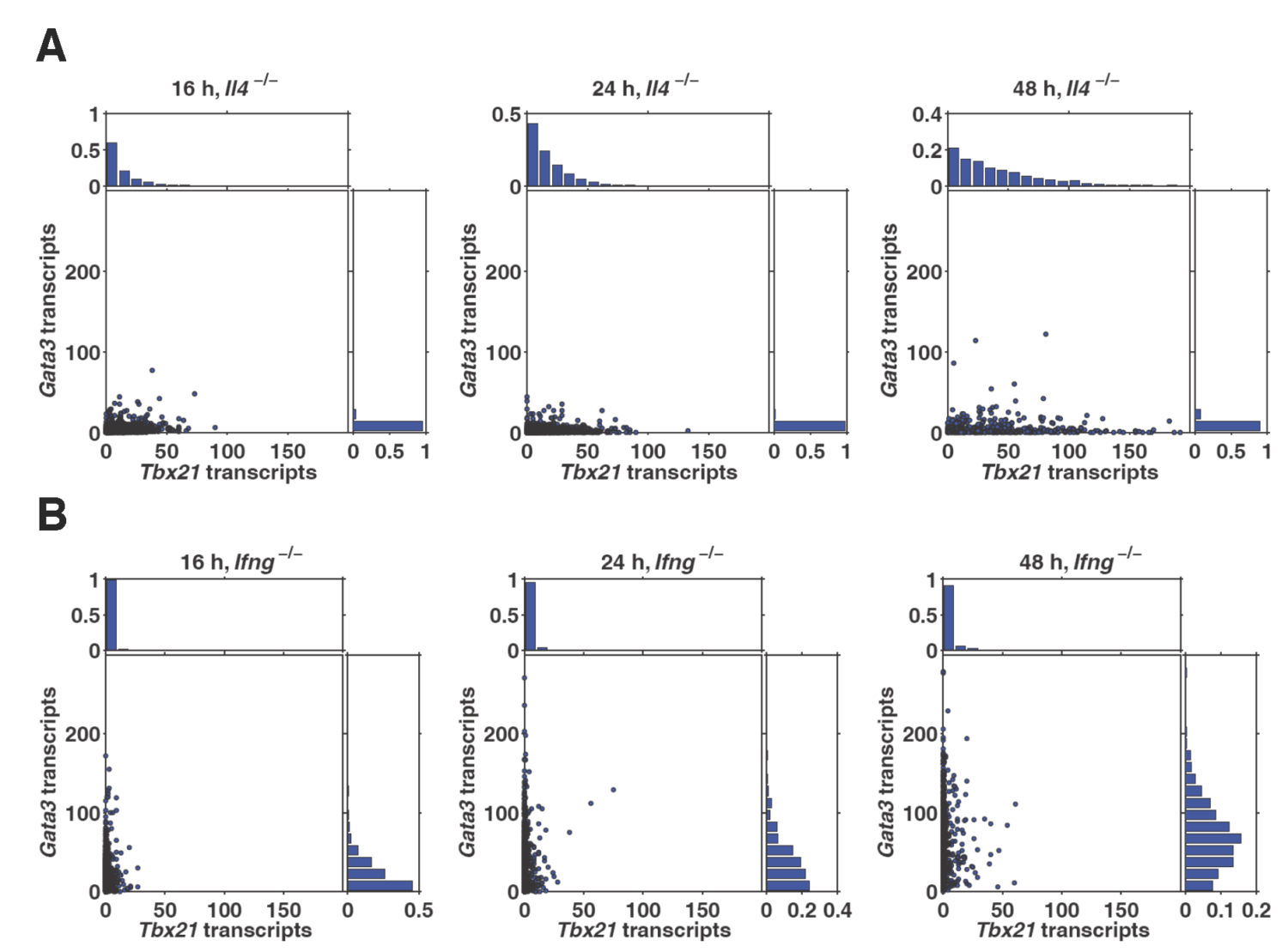

Supplement: Figure S7 — Scatter plots of Tbx21 and Gata3 transcripts in individual cells of Il4−/− (A) and Ifng −/− (B) mice, with marginal distributions at 16 h, 24 h, and 48 h. The expression of Gata3 is down-regulated in Il4−/− mice. The expression of Tbx21 is down-regulated in Ifng−/− mice. (TIF) [file pbio.1001618.s007.tif]

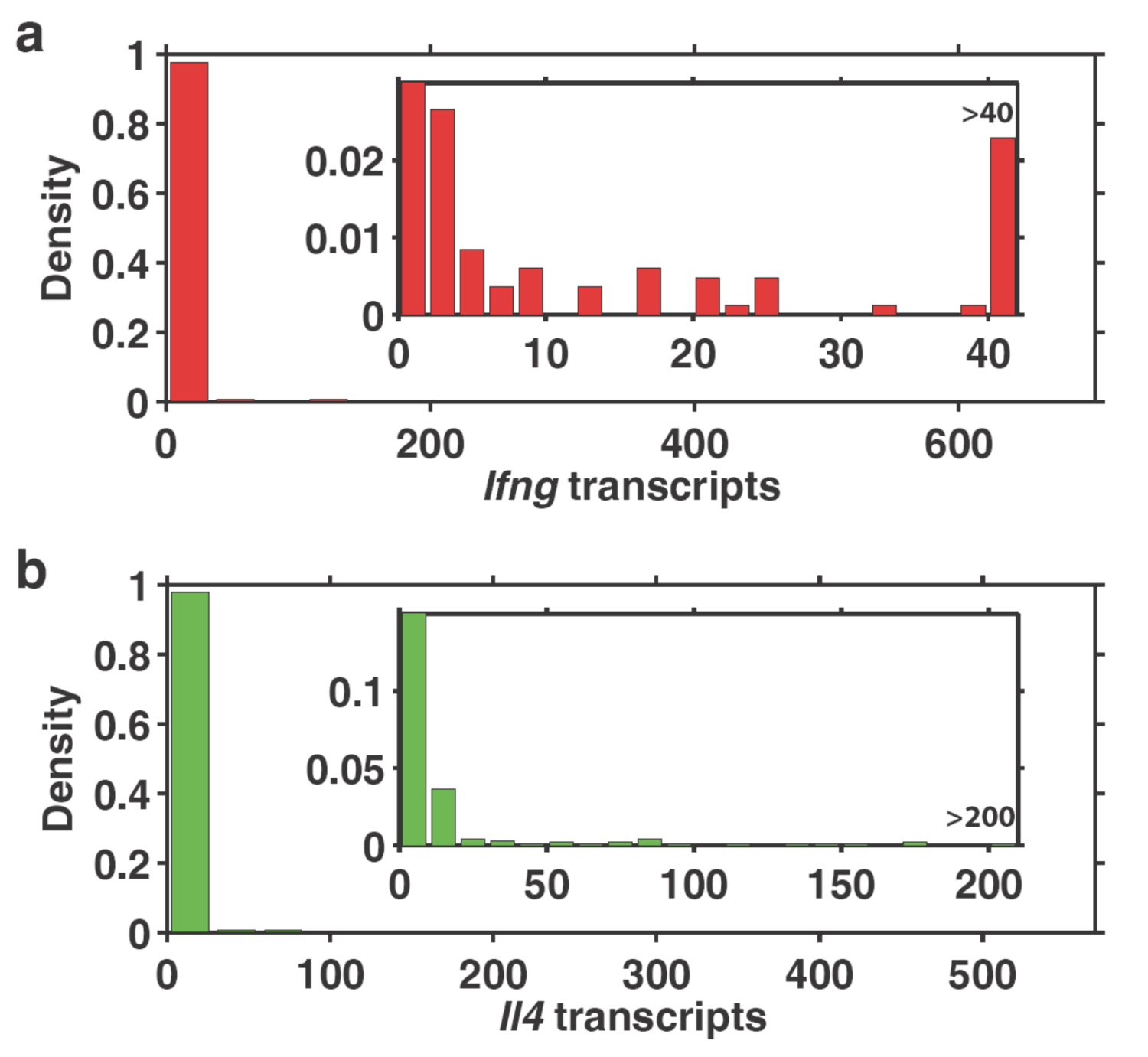

Supplement: Figure S8 — Fraction of cytokine-expressing cells at 24 h, in a control experiment that uses CD4 T cells purified by negative selection (MACS CD4+ T cell isolation kit II), in contrast to CD4 T cells purified by positive selection by CD4+ microbeads used in all the other experiments in this study. Panel (a) shows the probability density of cells expressing Ifng transcripts; panel (b) shows the probability density of cells expressing Il4 transcripts. We have shown that cultures of cells selected by negative selection also give rise to rare cells that stochastically express Ifng and Il4 at high levels. Therefore, rare cytokine-expressing cells observed in Figure 3A,B are not an artifact of positive selection by CD4+ microbeads. (TIF) [file pbio.1001618.s008.tif]

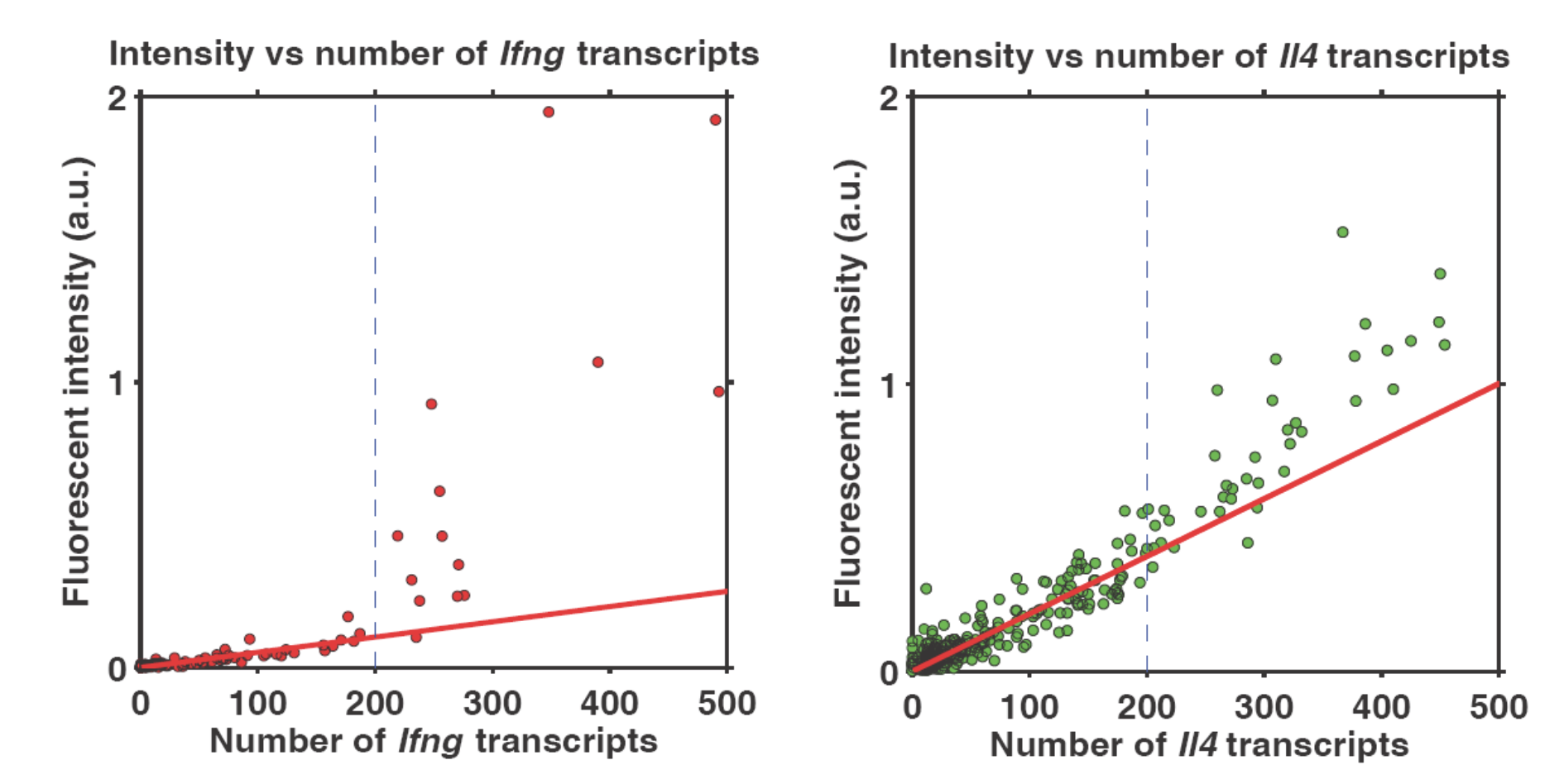

Supplement: Figure S9 — Linear relationship exists between total fluorescent intensity of FISH and the computed mRNA transcripts in cells expressing fewer than 200 transcripts. For the Ifng plot excluding points with more than 200 computed mRNA transcripts, Pearson's correlation coefficient = 0.86, ; for the Il4 plot excluding points with more than 200 computed mRNA transcripts, Pearson's correlation coefficient = 0.90, . We can then extrapolate of the number of transcripts in highly expressing cells using the slope of the linear fit for cells expressing fewer than 200 transcripts. (TIF) [file pbio.1001618.s009.tif]

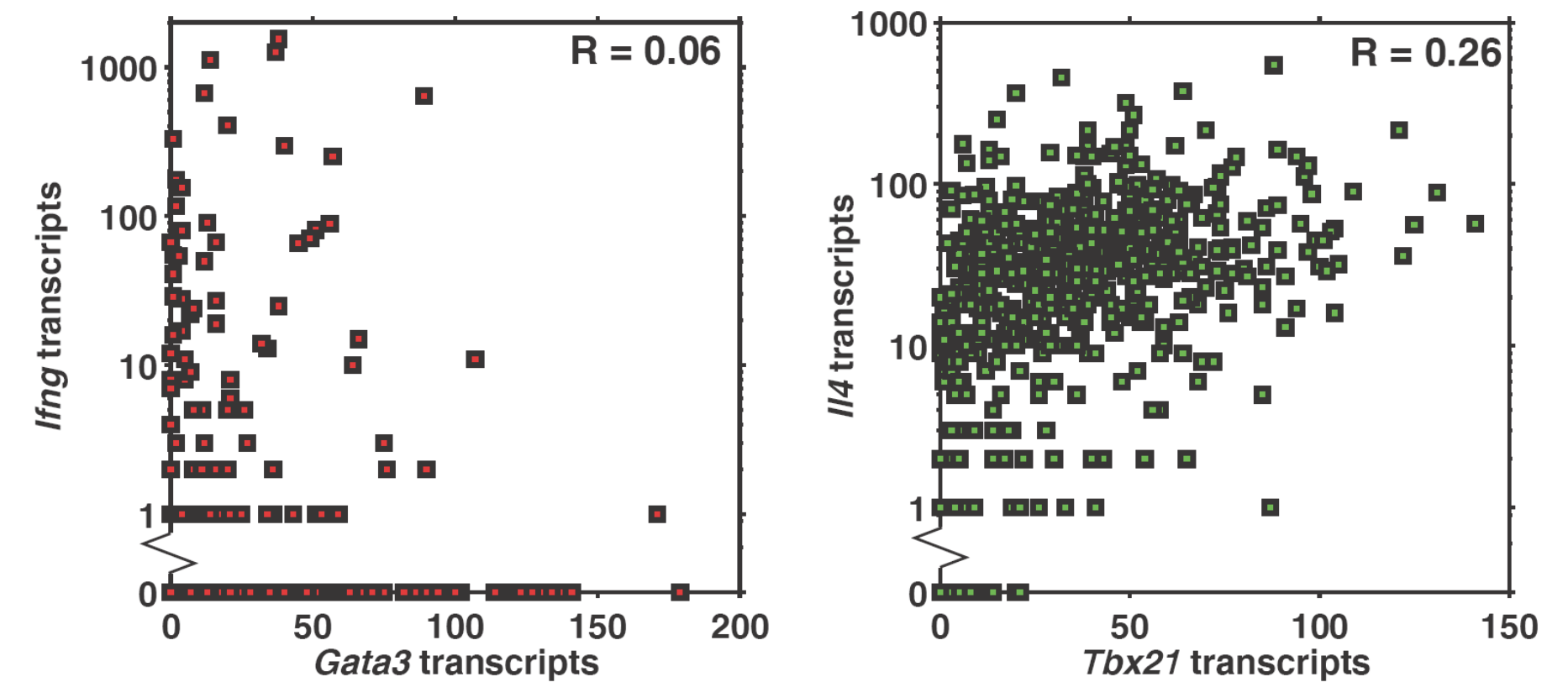

Supplement: Figure S10 — Scatter plots showing that there is no negative correlation between Gata3 and Ifng expression, with Pearson's correlation coefficient = 0.06, , and that there is no negative correlation between Tbx21 and Il4 expression, with Pearson's correlation coefficient = 0.26, . (TIF) [file pbio.1001618.s010.tif]

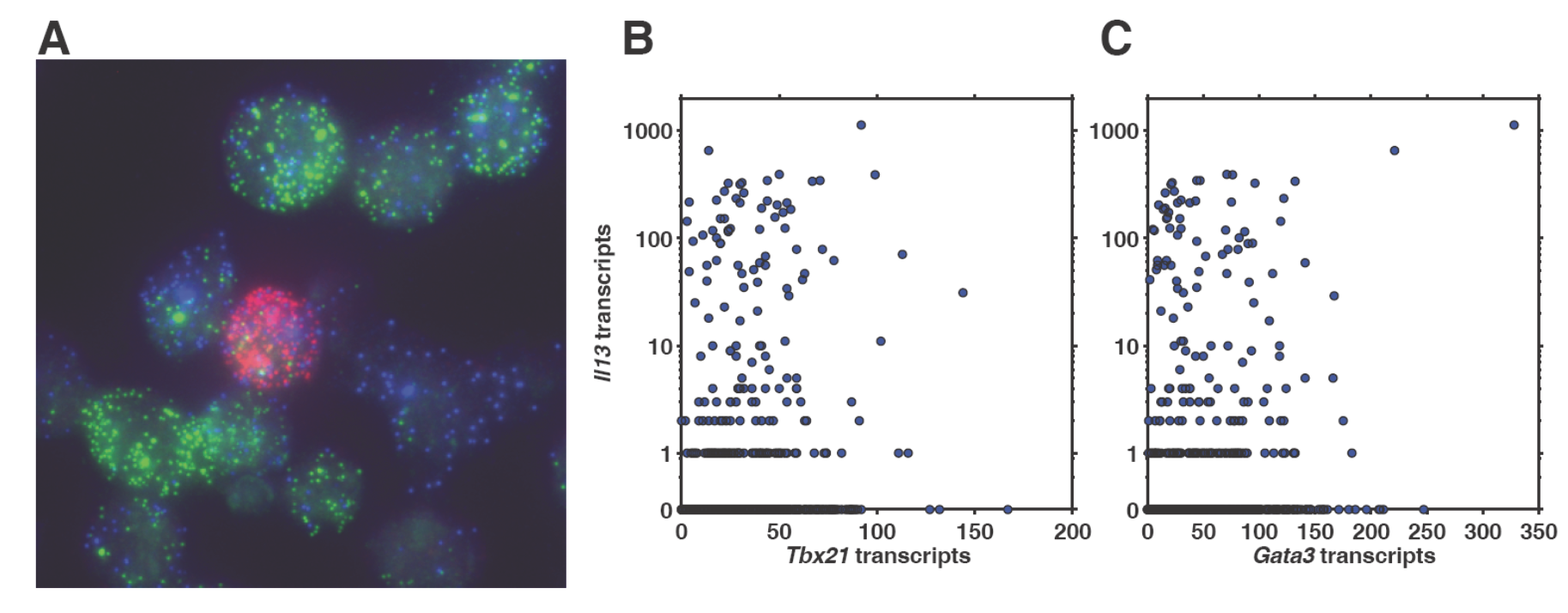

Supplement: Figure S11 — The expression of Il13 in activated Th cells has no strong correlation with the expression of Tbx21 or Gata3. (A) A fluorescent image of three-color smFISH probing Il13 (red), Tbx21 (blue), and Gata3 (green). (B) Scatter plot of the number of Il13 transcripts versus Tbx21 in CD4 T cells at 48 h with a Pearson's correlation coefficient R of 0.098 (). (C) Scatter plot of the number of Il13 transcripts versus Gata3 in CD4 T cells at 48 h with a Pearson's correlation coefficient R of 0.19 (). (TIF) [file pbio.1001618.s011.tif]

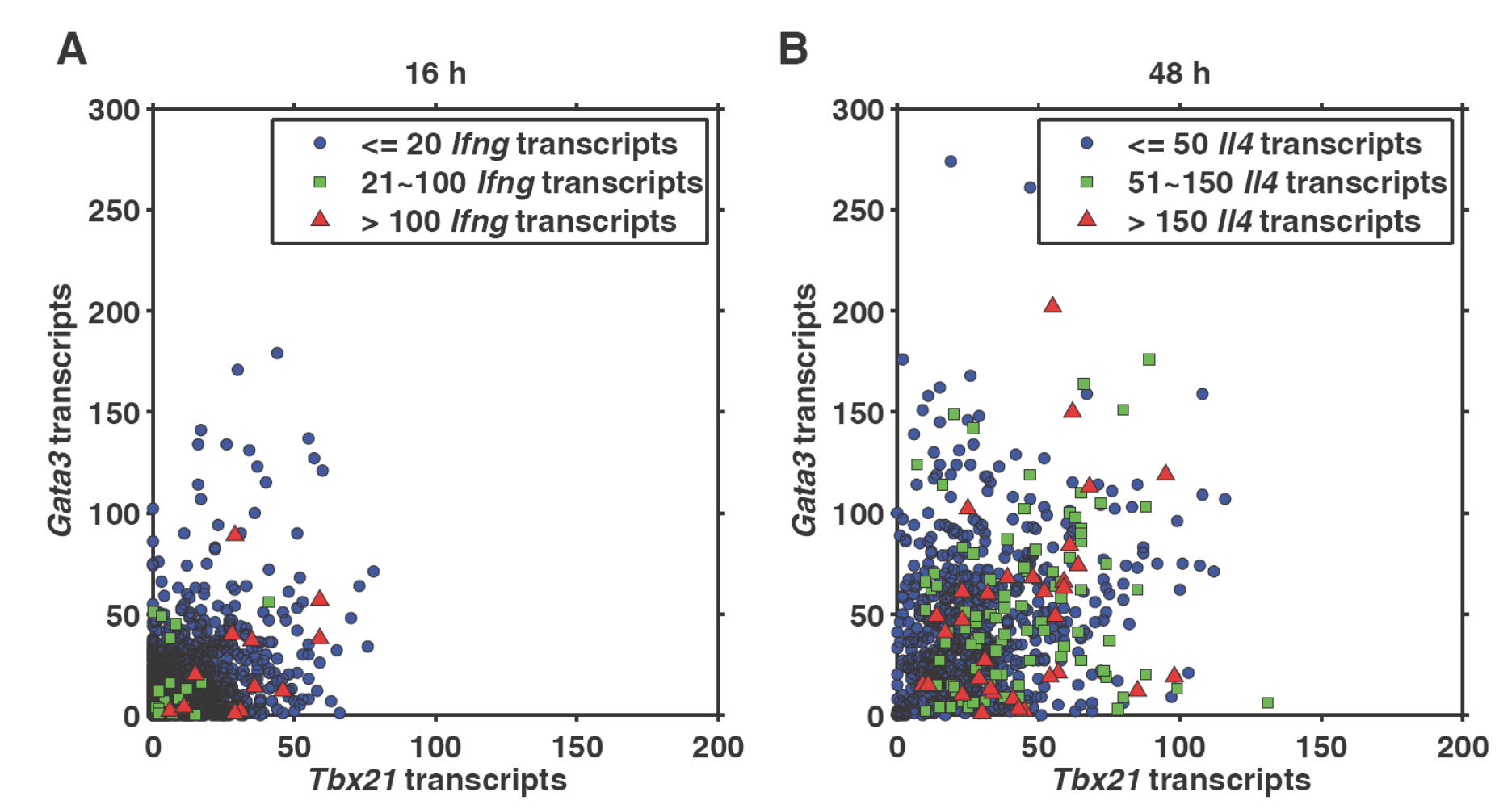

Supplement: Figure S12 — The expression of Tbx21 and Gata3 does not depend on the expression of cytokines. (A) Scatter plot of Tbx21 versus Gata3 color coded based on the expression of Ifng. (B) Scatter plot of Tbx21 versus Gata3 color coded based on the expression of Il4. (TIF) [file pbio.1001618.s012.tif]

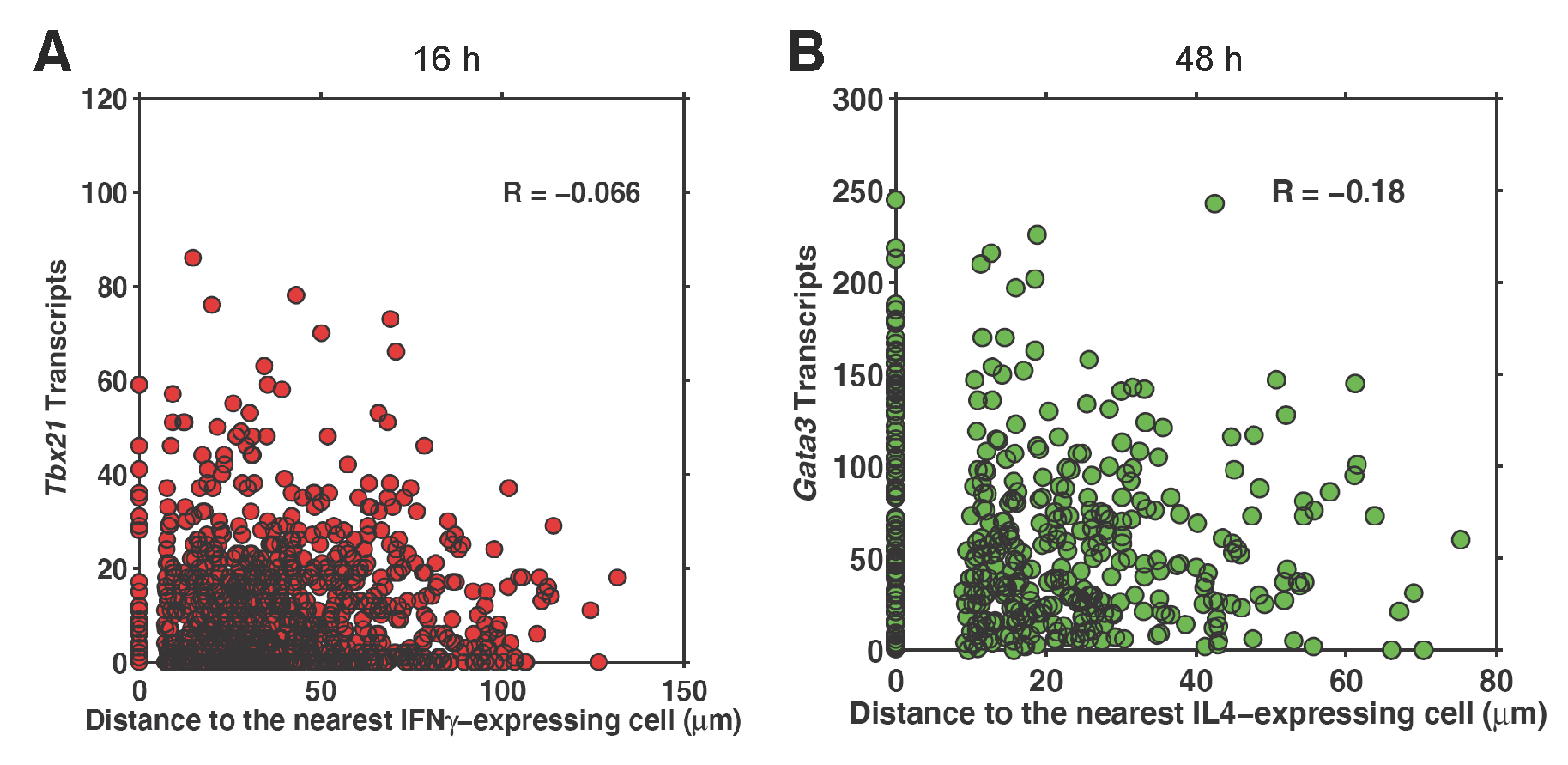

Supplement: Figure S13 — The scatter plot of Tbx21 (A) and Gata3 (B) transcripts in individual cells versus the distance to the nearest Ifng -expressing (A) or Il4 -expressing cell (B), which is defined as containing more than 20 transcripts of cytokines. The position of each cell is computed as its centroid. It shows that the expression level of Tbx21 and Gata3 does not correlate with the distance from the near cytokine-expressing cell. Therefore, diffusion of cytokines from the source cells is not rate limited on the time scale of Tbx21 and Gata3 expression. Note that cells at 0 µm for the distance axis are the cytokine-expressing cells. Absence of cells between 0 µm and 7 µm is attributed to the fact that cell diameter is 7 µm, because cells are not overlapping in the mono-layer for imaging. (TIF) [file pbio.1001618.s013.tif]

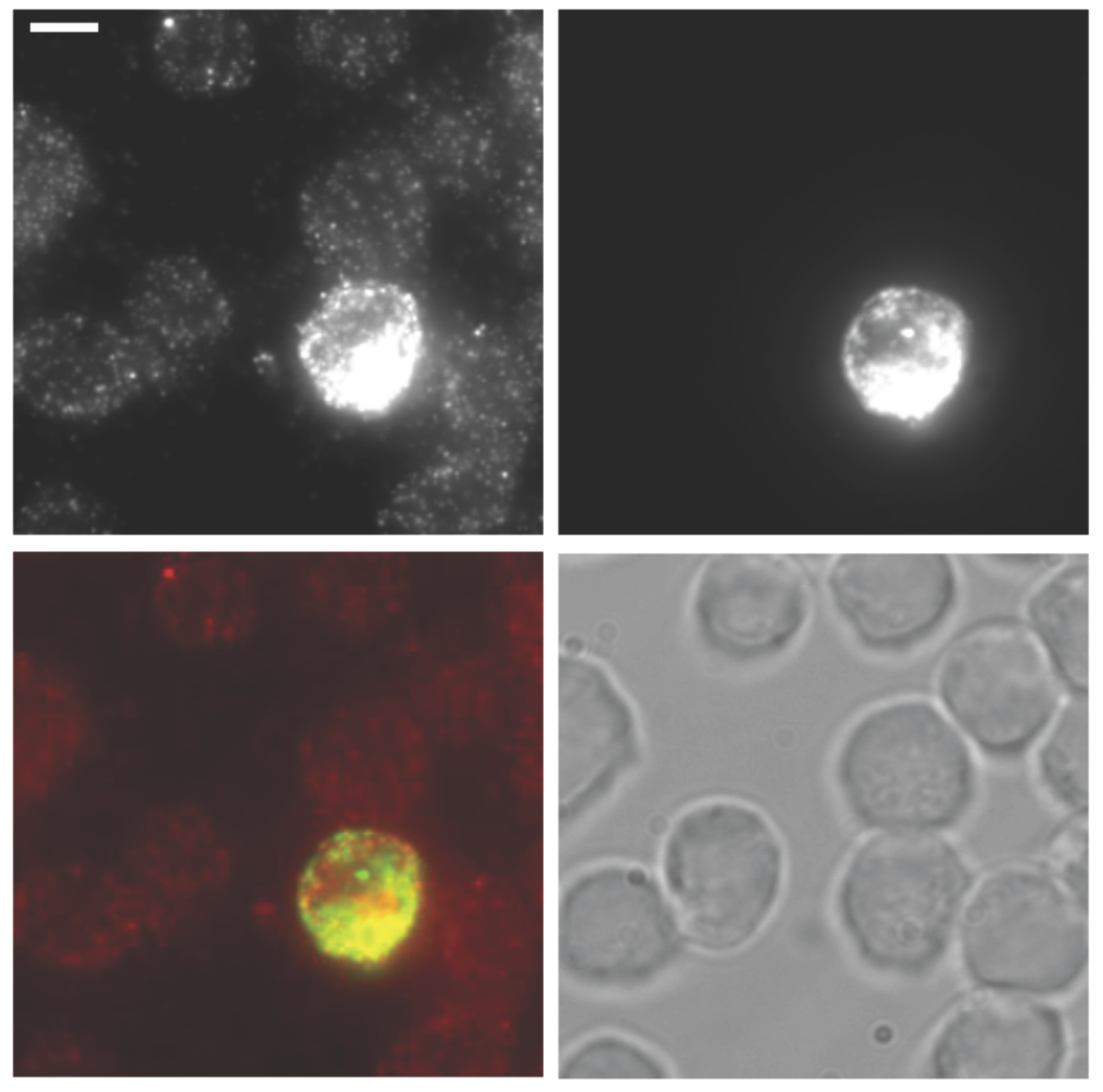

Supplement: Figure S14 — Immunofluorescence together with single-molecule FISH on IFNγ shows that only cells expressing Ifng transcripts contain IFNγ protein. Cytokine secretion was inhibited for 1 h to allow cytokine accumulation in these cells before harvesting. The top left panel is immunofluorescence image; the top right panel is single-molecule FISH image; the bottom left panel is the merge of immunofluorescence and single-molecule FISH; the bottom right panel is the bright field image. Scale bar, 10 µm. (TIF) [file pbio.1001618.s014.tif]

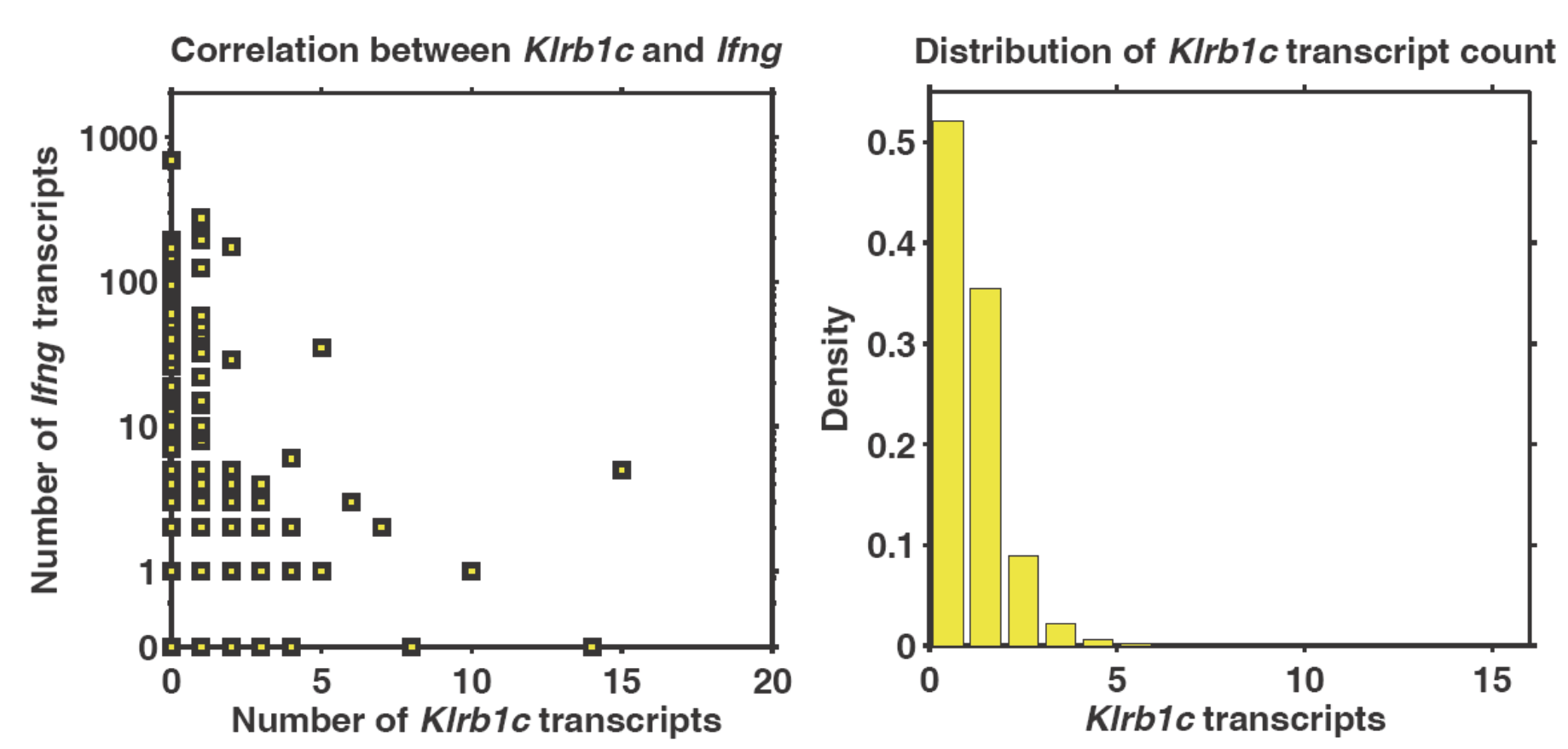

Supplement: Figure S15 — The cytokine-expressing cells are not NKT cells. The left panel is the scatter plot of Ifng and Klrb1c transcripts showing that there is no significant positive correlation between Ifng and Klrb1c, Pearson's correlation coefficient = 0.095, p = 0.001, at 16 h after activation; the right panel shows the distribution of Klrb1c transcripts, indicating that Klrb1c expression is essentially OFF in all cells. Because Klrb1c encodes the marker NK1.1 for NKT cells, the cells expressing Ifng are not NKT cells that are not removed during magnetic sorting. (TIF) [file pbio.1001618.s015.tif]

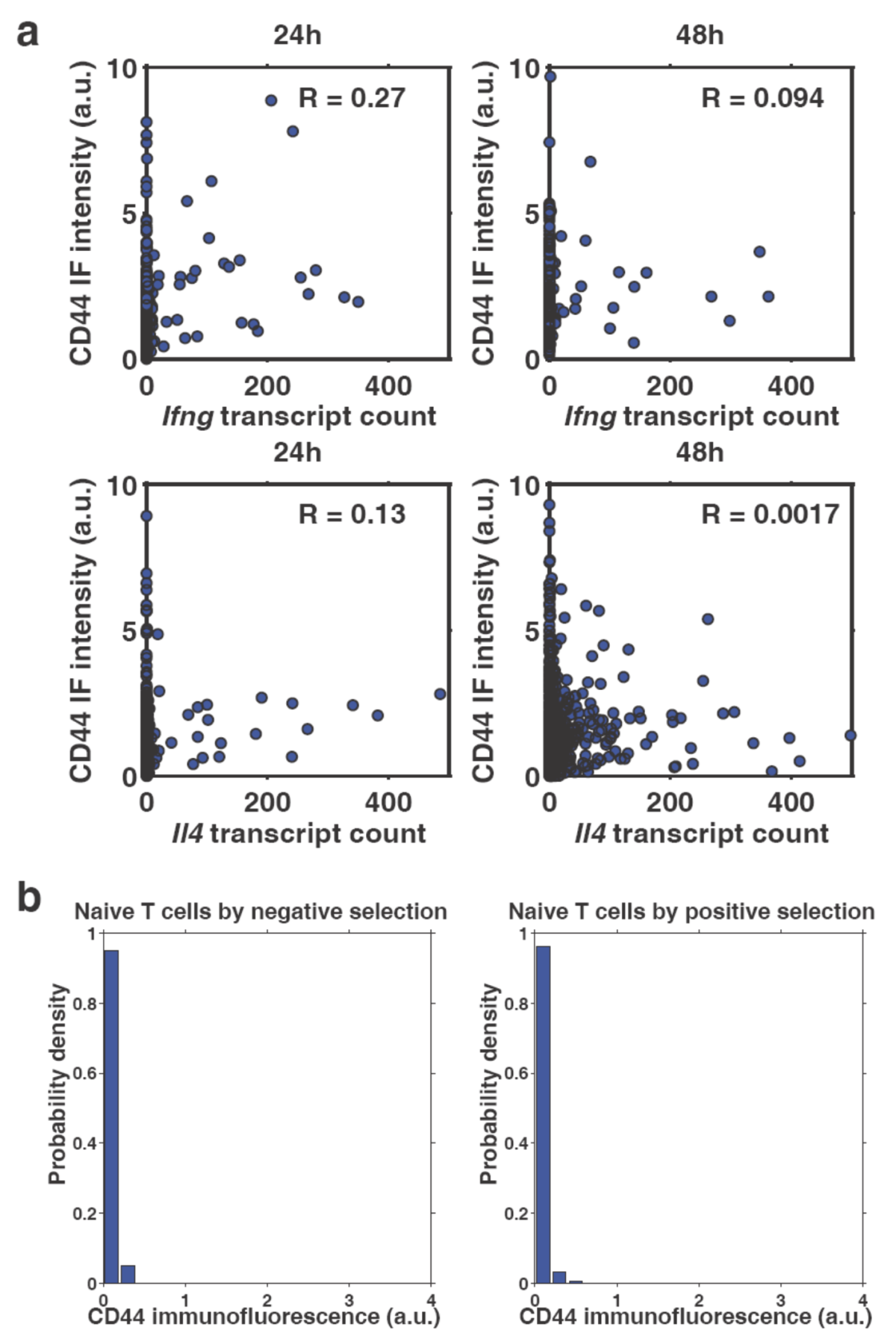

Supplement: Figure S16 — Cytokine-expressing cells are not memory T cells. (a) Scatter plot of CD44 immunofluorescence versus the number of Ifng or Il4 transcripts shows that there is no significant positive correlation between CD44 levels and Ifng (correlation coefficient = 0.27, at 24 h; correlation coefficient = 0.094, at 48 h) or Il4 expression (correlation coefficient = 0.13, at 24 h; correlation coefficient = 0.0017, at 48 h). Cd44 is a marker of memory T cells. Because cytokine-expressing cells do not preferentially express high levels of Cd44 transcripts, they are not contaminating memory T cells that are not removed during magnetic sorting. (b) Probability density plot of CD44 immunofluorescence of naive T cells isolated by positive selection (CD4+ microbeads) or depletion (MACS CD4+ T cell isolation kit II). It shows that T cells isolated by positive selection, as used ubiquitously in this paper, are similar to T cells isolated by depletion, have low CD44 levels, and do not contain memory cells that are CD44+. (TIF) [file pbio.1001618.s016.tif]

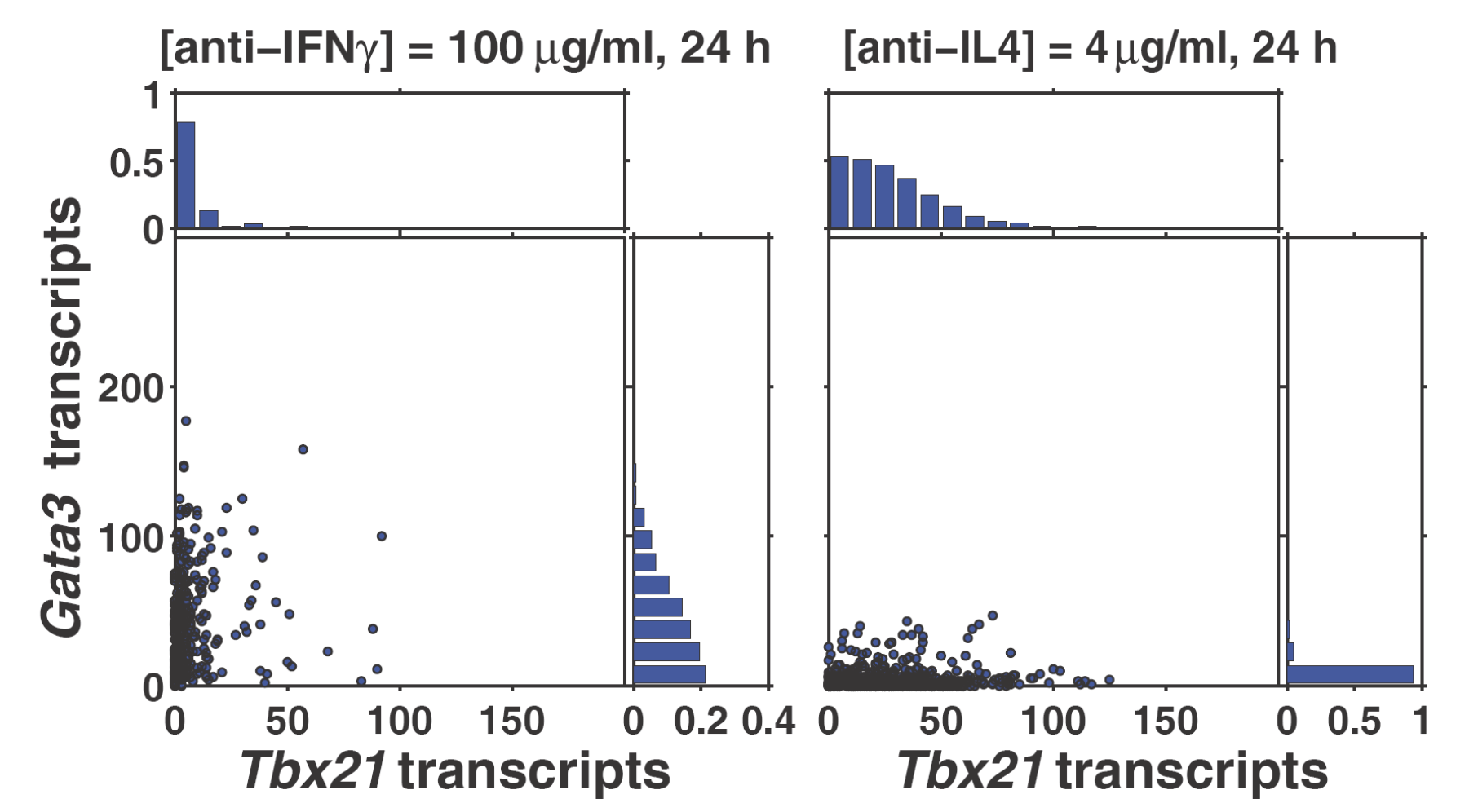

Supplement: Figure S17 — Scatter plots and marginal distributions showing that IFNγ antibody down-regulates Tbx21 and IL4 antibody down-regulates Gata3 at 24 h. (TIF) [file pbio.1001618.s017.tif]

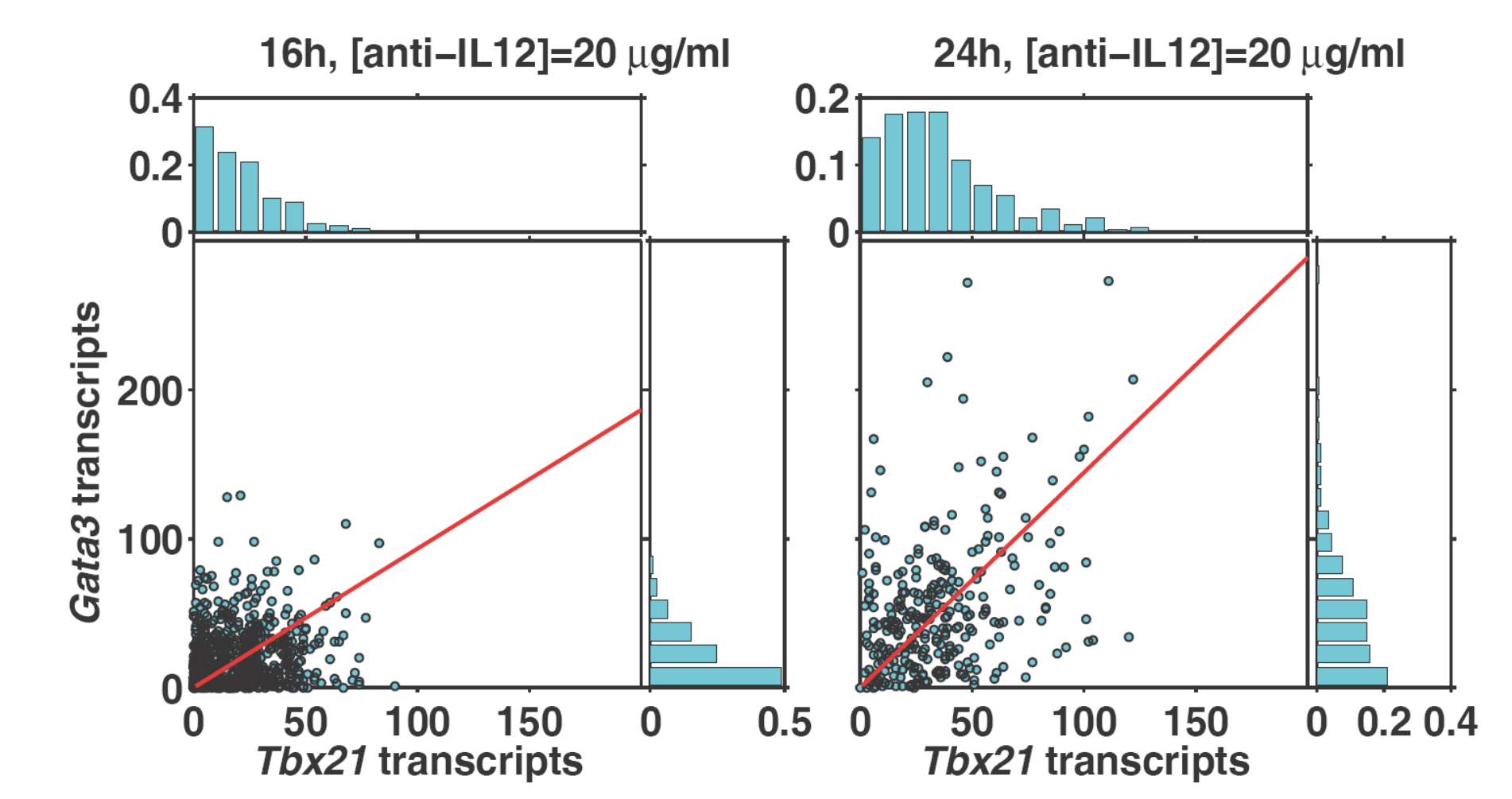

Supplement: Figure S18 — Scatter plots and marginal distributions of Tbx21 and Gata3 transcripts in individual cells treated with IL12 antibody, with the red line dividing data points into halves. The left panel shows cells 16 h after activation; the right panel shows cells 24 h after activation. The result shows that anti-IL12 has no effect on the expression of Tbx21 during early differentiation of Th cells. (TIF) [file pbio.1001618.s018.tif]

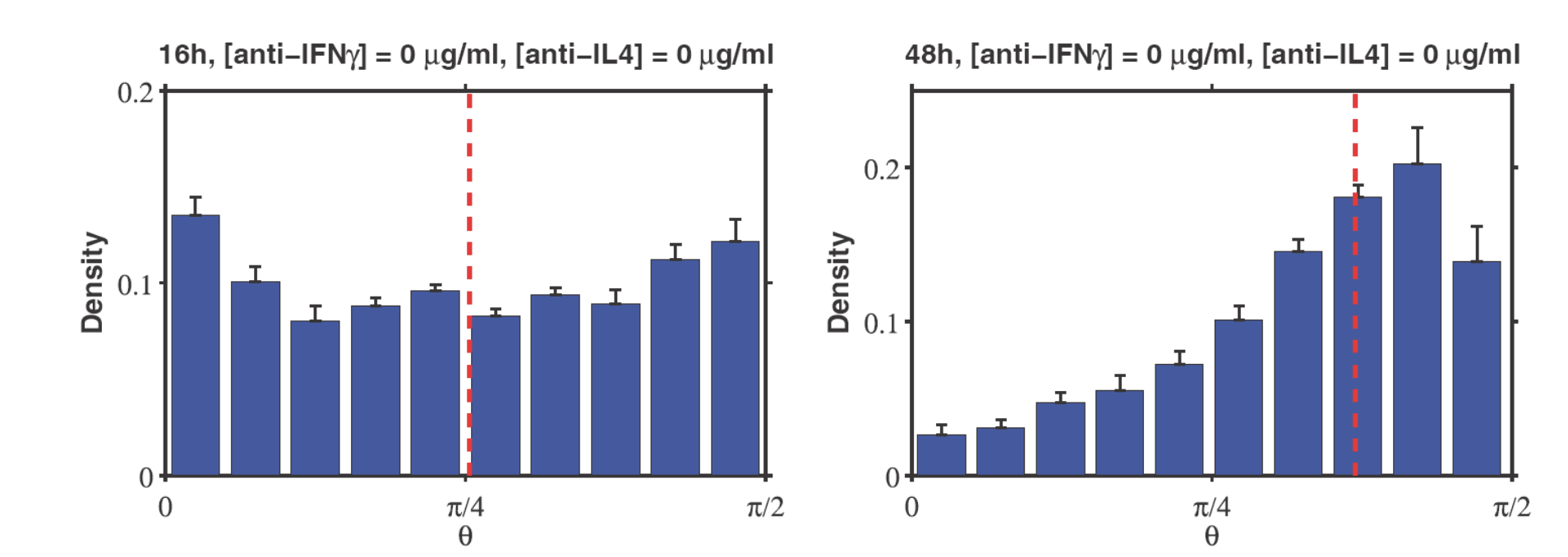

Supplement: Figure S19 — Distribution of θ under non-biased condition. The left panel is 16 h after activation, where θ follows a uniform distribution. The right panel is 48 h after activation, where θ is skewed toward , indicating cells become more Th2-like. (TIF) [file pbio.1001618.s019.tif]

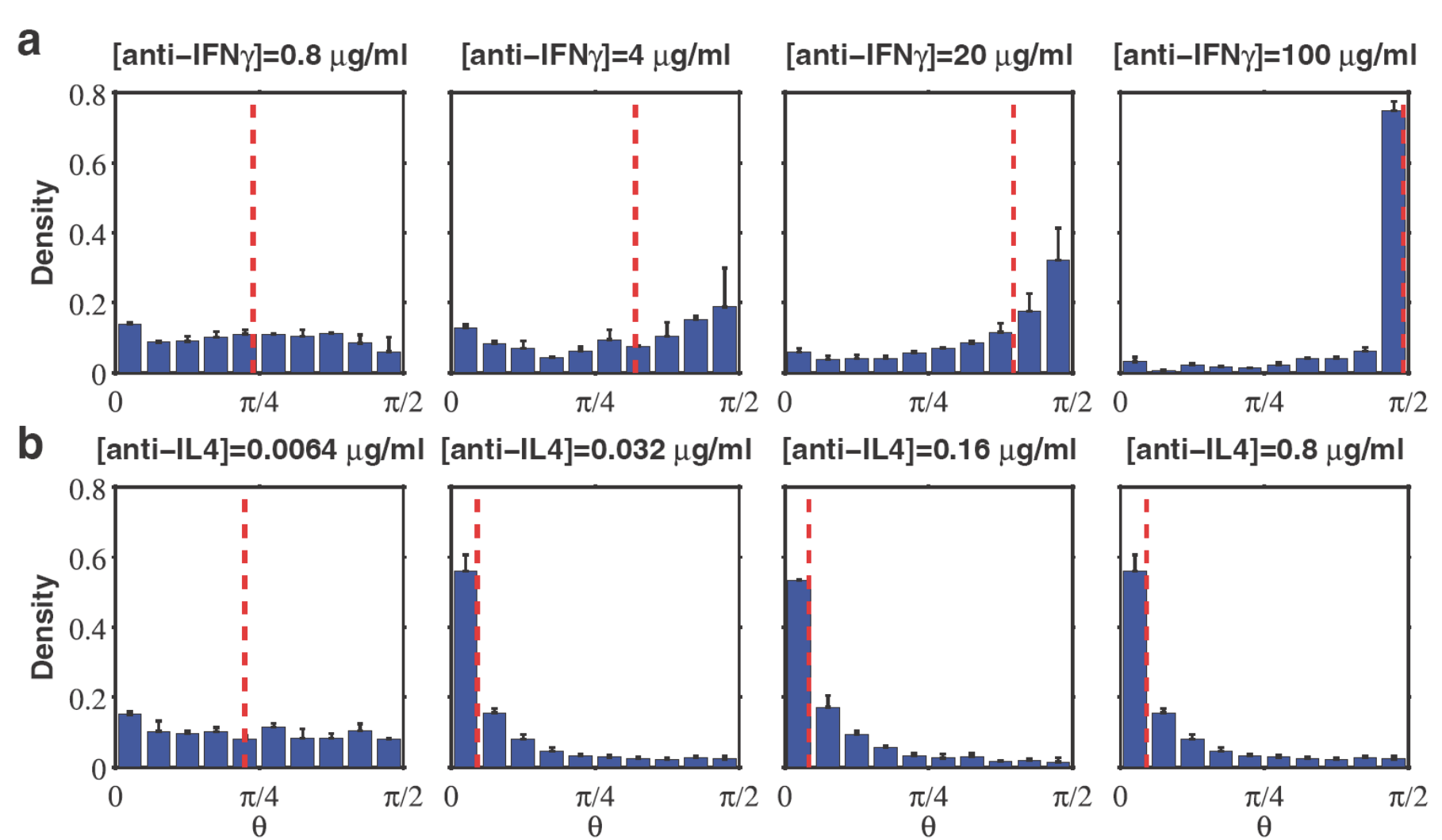

Supplement: Figure S20 — Distribution of θ at 16 h after activation. Panel (a) shows that as concentration of anti-IFNγ antibody increases, the cells adopt larger θ. Panel (b) shows that as concentration of anti-IL4 antibody increases, the cells adopt smaller θ. Red lines are the medians of the θ distribution. (TIF) [file pbio.1001618.s020.tif]

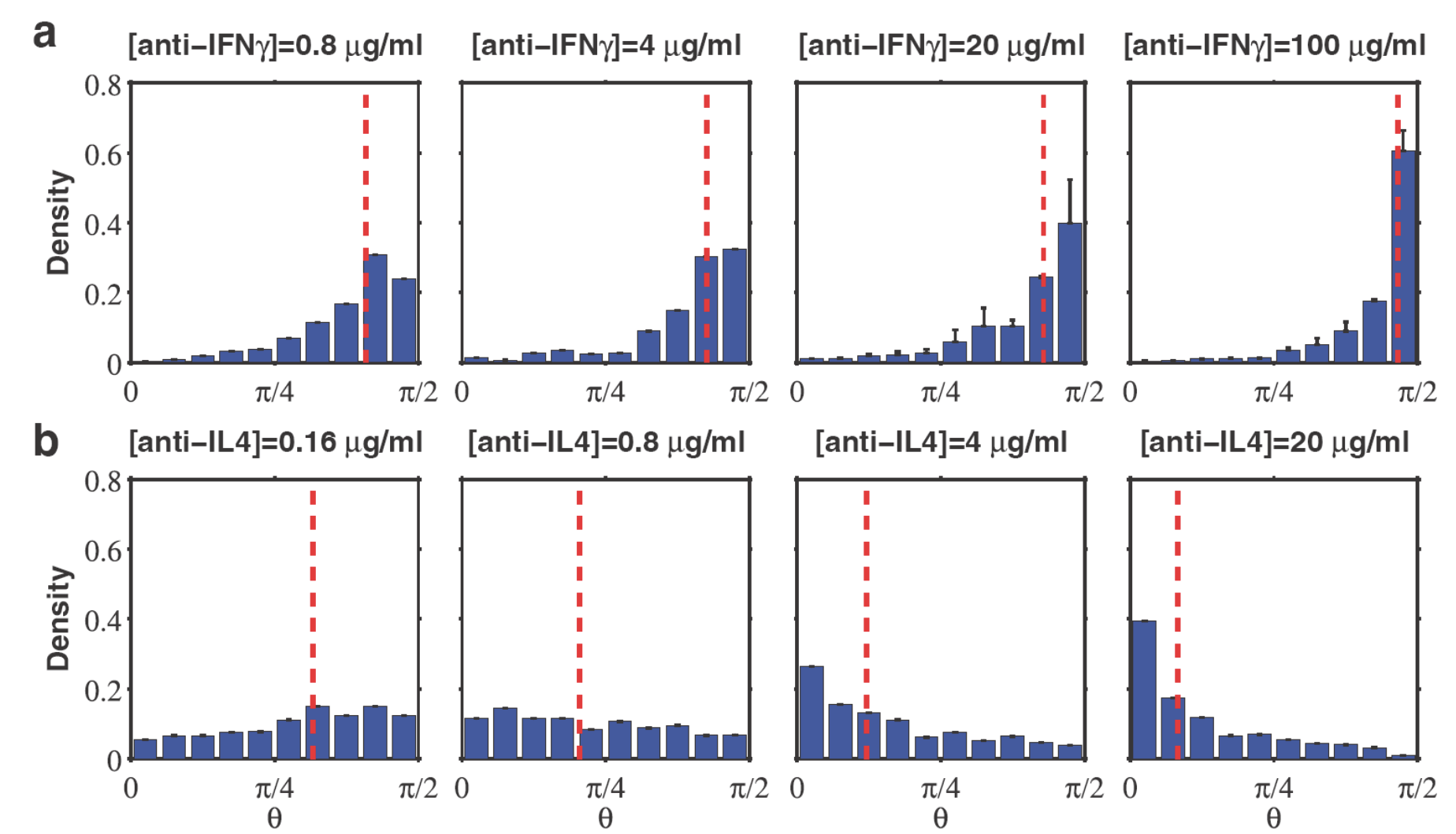

Supplement: Figure S21 — Distribution of θ at 48 h after activation. Panel (a) shows that as concentration of anti-IFNγ antibody increases, the cells adopt larger θ. Panel (b) shows that as concentration of anti-IL4 antibody increases, the cells adopt smaller θ. Red lines are the medians of the θ distribution. (TIF) [file pbio.1001618.s021.tif]

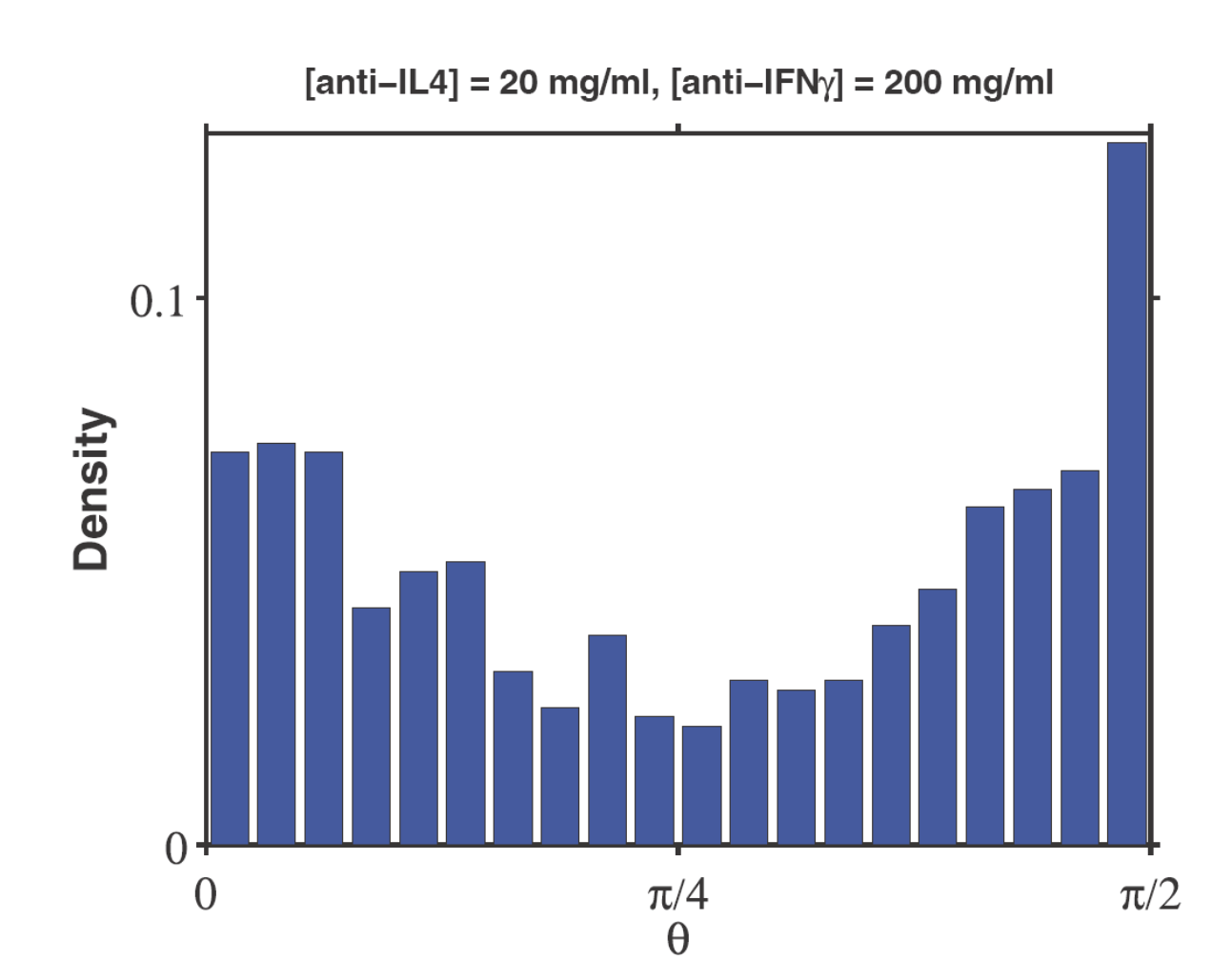

Supplement: Figure S22 — Distribution of θ at 48 h, where cells were not treated with any polarizing antibodies for the first 24 h, followed by the addition of both anti-IFNγ and anti-IL4 antibodies at 24 h. It shows that the vast majority of cells adopt either very large or small θ, adopting either a Th1-like or Th2-like cell fate. (TIF) [file pbio.1001618.s022.tif]

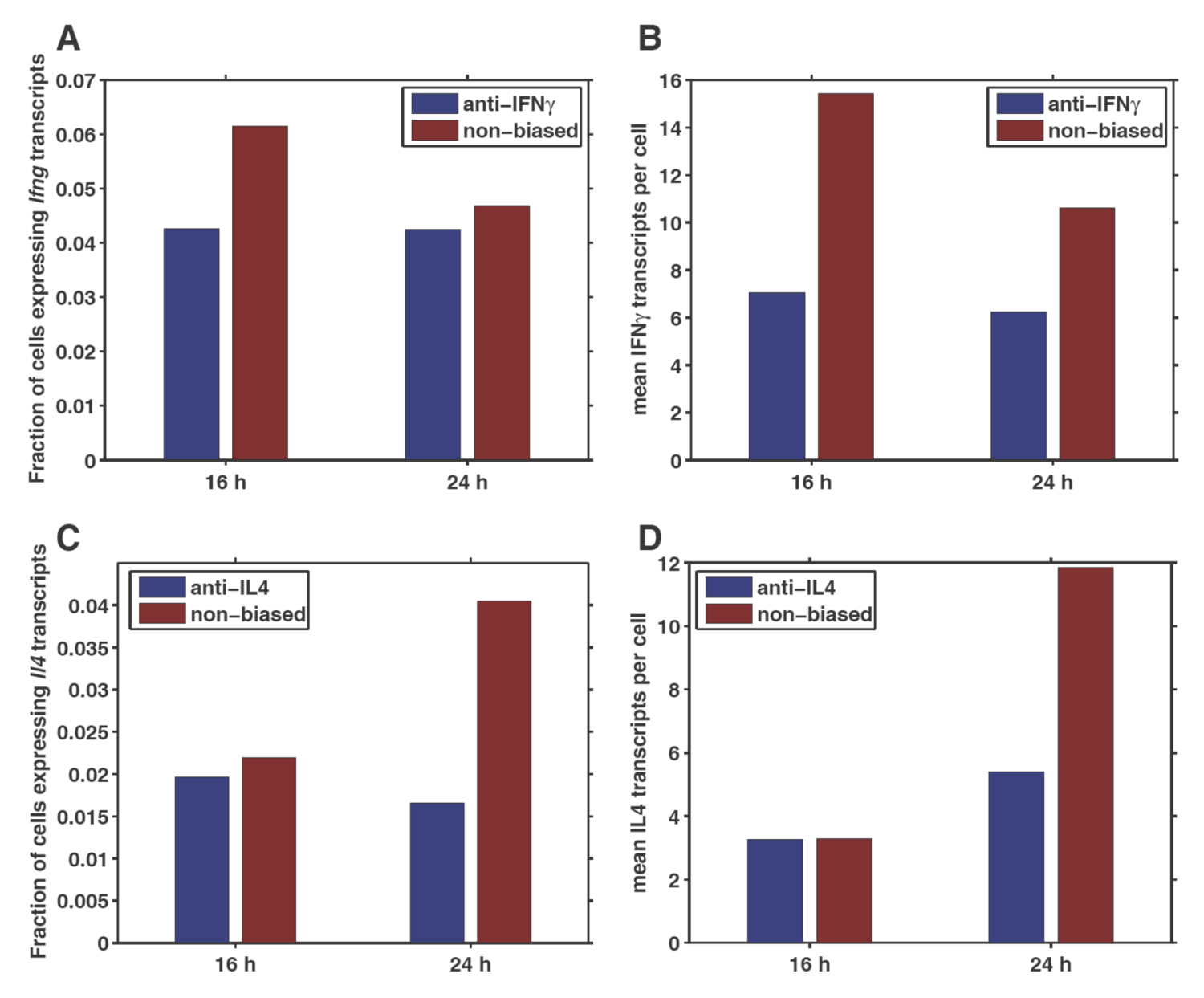

Supplement: Figure S23 — Cytokine expression is down-regulated in the presence of neutralizing antibodies. (A) Fraction of Ifng-expressing (defined as having >20 transcripts) decreases when anti-IFNγ is present in the cell culture. (B) Mean number of Ifng transcript per cell (defined as having >20 transcripts) decreases when anti-IFNγ is present in the cell culture. (C) Fraction of Il4-expressing (defined as having >50 transcripts) decreases when anti-IL4 is present in the cell culture. (D) Mean number of Il4 transcript per cell (defined as having >50 transcripts) decreases when anti-IL4 is present in the cell culture. (TIF) [file pbio.1001618.s023.tif]
